# Huntington disease oligodendrocyte maturation deficits revealed by single-nucleus RNAseq are rescued by thiamine-biotin supplementation

**DOI:** 10.1038/s41467-022-35388-x

**Published:** 2022-12-21

**Authors:** Ryan G. Lim, Osama Al-Dalahmah, Jie Wu, Maxwell P. Gold, Jack C. Reidling, Guomei Tang, Miriam Adam, David K. Dansu, Hye-Jin Park, Patrizia Casaccia, Ricardo Miramontes, Andrea M. Reyes-Ortiz, Alice Lau, Richard A. Hickman, Fatima Khan, Fahad Paryani, Alice Tang, Kenneth Ofori, Emily Miyoshi, Neethu Michael, Nicolette McClure, Xena E. Flowers, Jean Paul Vonsattel, Shawn Davidson, Vilas Menon, Vivek Swarup, Ernest Fraenkel, James E. Goldman, Leslie M. Thompson

**Affiliations:** 1 UCI MIND, University of California Irvine, Irvine, CA USA; 2 Department of Pathology and Cell Biology, Vagelos College of Physicians and Surgeons, Columbia University Irving Medical Center, New York, NY USA; 3 Department of Biological Chemistry, University of California Irvine, Irvine, CA USA; 4 Department of Biological Engineering, Massachusetts Institute of Technology, Cambridge, MA USA; 5 Department of Neurology, Vagelos College of Physicians and Surgeons, Columbia University Irving Medical Center, New York, NY USA; 6 Advanced Science Research Center at the City University of New York, New York, NY USA; 7 Department of Psychiatry and Human Behavior, University of California Irvine, Irvine, CA USA; 8 Department of Neurobiology and Behavior, University of California Irvine, Irvine, CA USA; 9 Department of Pathology, University of California Irvine, Irvine, CA USA; 10 Taub Institute for Research on Alzheimer’s Disease and the Aging Brain, Columbia University Irving Medical Center, New York City, New York, NY USA; 11 Lewis-Sigler Institute for Integrative Genomics, Princeton, NJ USA; 12 Sue and Bill Gross Stem Cell Center University of California Irvine, Irvine, CA USA

**Keywords:** Huntington's disease, Huntington's disease, Mechanisms of disease

## Abstract

The complexity of affected brain regions and cell types is a challenge for Huntington’s disease (HD) treatment. Here we use single nucleus RNA sequencing to investigate molecular pathology in the cortex and striatum from R6/2 mice and human HD post-mortem tissue. We identify cell type-specific and -agnostic signatures suggesting oligodendrocytes (OLs) and oligodendrocyte precursors (OPCs) are arrested in intermediate maturation states. OL-lineage regulators *OLIG1* and *OLIG2* are negatively correlated with CAG length in human OPCs, and ATACseq analysis of HD mouse NeuN-negative cells shows decreased accessibility regulated by OL maturation genes. The data implicates glucose and lipid metabolism in abnormal cell maturation and identify *PRKCE* and Thiamine Pyrophosphokinase 1 (*TPK1*) as central genes. Thiamine/biotin treatment of R6/1 HD mice to compensate for *TPK1* dysregulation restores OL maturation and rescues neuronal pathology. Our insights into HD OL pathology spans multiple brain regions and link OL maturation deficits to abnormal thiamine metabolism.

## Abstract (web summary)

Here the authors evaluate single cell gene expression from mouse and human Huntington’s disease brains, finding incomplete oligodendrocyte maturation and pathways involved. Treating mice with thiamine/biotin ameliorates molecular pathology.

## Introduction

Huntington disease (HD) is a progressive neurodegenerative disease characterized by prominent loss of medium spiny neurons (MSN) in the striatum and cortical atrophy^1^. The disease, which manifests with cognitive, psychiatric and movement impairments, is caused by an autosomal dominant CAG repeat expansion in the first coding exon of the Huntingtin gene and a corresponding expanded polyglutamine repeat in the Huntingtin (HTT) protein^2^. Genome-wide approaches, including bulk RNA- and ChIP-sequencing, have facilitated understanding the molecular impact of mutant HTT (mHTT) expression in a variety of model systems^3–6^ and have suggested deficits in neurodevelopmental programs in HD^3,7–9^. However, bulk tissue analysis limits the understanding of cell type-specific changes. The ability to distinguish common signatures of HD across multiple cell types from those that are unique to specific cell types can facilitate our mechanistic understanding of the disease. Past studies uncover these differences by expressing mHTT using cell type-specific drivers in animal models of HD^10^ or using human HD induced pluripotent stem cells (iPSCs) differentiated to specific cell types; these studies support the idea that cell type-specific effects of HD synergistically lead to pathogenesis^11,12^. Recent studies using single cell transcriptomics approaches also show cell type-specific neurodevelopmental impairments in HD^13,14^.

One such cell type are oligodendrocytes (OL) for which there has been a growing awareness that these and other OL-lineage cells, including oligodendrocyte progenitor cells (OPCs), are abnormal in HD. Prior studies focusing on HD models have shown early myelination deficits based on structural and transcriptomic data in mouse models of HD^15,16^, and that OL targeted mHTT expression causes HD symptoms as well as myelination deficits and altered OL maturation in mice via a mechanism involving myelin regulatory factor (Myrf)^17^. Myelination deficits were also evident in BACHD and R6/2 mice^18,19^ and OL maturation impairments, glial dysfunction^20,21^ and impaired OPC differentiation have been described in HD. For example, HD embryonic stem cell-derived glial progenitors transplanted into shiverer mice exhibit decreased differentiation and hypomyelination^22^, while another study showed that remyelination was impaired in cuprizone-treated mice, implicating abnormal OPC function in HD^23^, and inactivation of mHTT in OPCs prevented myelin abnormalities in HD mice^11^. Corroborating these findings in HD models, several studies have shown myelination and OL impairment in HD patient tissue. Bulk transcriptional studies of HD postmortem tissues revealed that *MYT1L*, a myelin transcription factor, and *MBP* were decreased in the caudate and prefrontal cortex, respectively^24,25^, and radiographic and neuropathological studies also reveal that OLs and myelination are abnormal (summarized in ref. 26). Neuropathologic examination of postmortem HD brains revealed a higher density than normal of OLs in the caudate nucleus^27,28^, including in pre-symptomatic HD patients. Stereological examinations of white matter revealed a decrease of 20–30% of the cross-sectional area of white matter in coronal levels from frontal to occipital regions^29^, as well as in the fornix^30^, in both lower and higher HD grades, suggesting that white matter loss represents an early change.

To further support these prior findings of OL maturation deficits, provide additional insight into cell type-specific versus agnostic signatures, and fill in the gaps in knowledge of key drivers that regulate OL impairments, we used single nucleus-RNAseq (snRNAseq) to obtain cell type-specific gene expression data across multiple brain regions from both the rapidly progressing R6/2 mouse model^31^ and human post-mortem brain samples of increasing grades of disease severity—including both adult- and juvenile-onset HD—and used these data for correlative and causal network modeling. We detail the differences in cell type-specific and agnostic gene expression changes, as well as putative causal drivers of cell type-specific transcriptomic changes. Consistent with previous literature, we find that OL-lineage cells show significant transcriptional dysregulation. We expand on these observations, finding that HD OPCs and OLs have altered the expression of development and maturation genes in both mice and human, with many HD OL-lineage cells showing intermediate states of development. Causal network modeling identified putative key drivers whose expression and extent of dysregulation was shown to correlate with the CAG repeat length in human tissue. We found that a gene central to the OPC/OL causal network, Protein kinase C epsilon (*PRKCE*), was downregulated in human and mouse, and we provide functional studies to clarify its role in promoting OL maturation. These findings are further supported by evidence from ATACseq and subsequent validation studies. We also identified impairments in glucose and lipid metabolism, identified as cell type-agnostic signatures, as potential drivers of this pathology. This connection to metabolism led us to find potentially unique roles for diacylglycerol (DAG), which regulates PRKCE, and for thiamine and biotin (T&B) metabolic processes in HD OL maturation impairments. Thiamine Pyrophosphokinase 1 (*Tpk1)*, which converts thiamine into thiamine pyrophosphate, was differentially expressed in the most cell types in the 12w R6/2 mice, and both TPK1 and SLC19A2, a thiamine transporter, were downregulated in human HD. Interestingly, mutations in *TPK1* or the thiamine-transporter *SLC19A3* lead to thiamine pyrophosphate deficiencies and early-onset neurodegeneration with brain atrophy, basal ganglia impairment, and motor dysfunction which can be effectively treated with high dose T&B^32,33^. In addition, mutations in *SLC19A2* lead to Roger’s syndrome, with megaloblastic anemia, thrombocytopenia, diabetes mellites, and sensorineural deafness^34^ and general dietary thiamine deficiencies are known to contribute to a number of neurological and psychiatric symptoms^35^. To further examine potential connections between early metabolic changes in HD and OL maturation, we treated R6/1 mice, which has a longer therapeutic window than R6/2 mice and also show dys-maturation signatures in a number of cell types^14^, with T&B and conducted snRNAseq on the striata of T&B treated and vehicle-treated mice. T&B treatment resulted in significant rescue of dys-maturation signatures in OL and neurons, and an overall decrease in the number of significant differentially expressed genes (DEGs). Our data provide evidence that dysregulated metabolism and metabolic genes can directly contribute to the cell maturation deficits observed in OLs and other cell types, and that diet supplementation may be a therapeutic modality for HD.

## Results

### Single nuclei RNAseq of R6/2 mouse model of HD

**Fig. 1:**
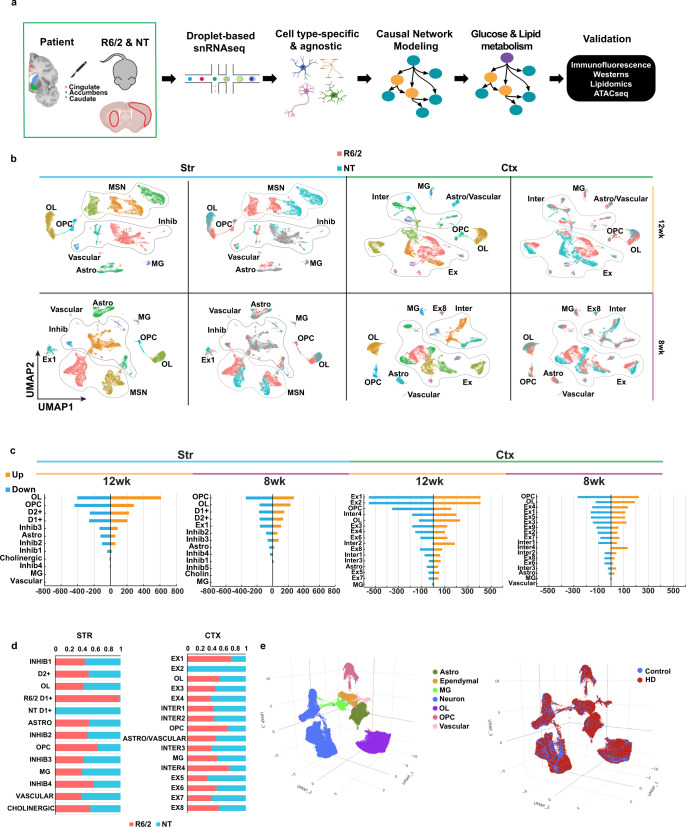
Single nucleus RNAseq of mouse and human R6/2 and HD samples. **a** Illustration of workflow used for this study. After frozen tissue is microdissected from the Cingulate, Caudate, and nucleus Accumbens from 66 samples from 29 human donors (3 grade I, 4 grade II, 4 grade III, 3 grade IV, 5 juvenile-onset HD, and 10 matched controls), or the striatum and cortex of the mice (*n* = 3), nuclei are isolated, 10× Libraries are prepared followed by next generation sequencing. Created with BioRender.com **b** Uniform manifold projection and approximation plots (UMAP) of the R6/2 and NT mouse data colored by cluster or genotype. Initial QC and filtering led to the identification of 108,974 nuclei from mouse tissues. General cell type annotations: Astro Astrocytes, OL Oligodendrocyte, OPC Oligodendrocyte progenitors, MSN Medium spiny neurons, Inhib inhibitory neurons, MG Microglia, Ex Excitatory neurons, Inter Interneurons. **c** Barplot showing the number of up (orange) and down (blue) regulated DEGs per a cell type in the mouse data. **b**, **c** Striatal (Str, light blue bar) samples on the left and cortical (Ctx, light green bar) samples on the right, 12w samples marked by yellow bar and 8w marked by purple bar. **d** Proportion of R6/2 and NT cells within each cluster, red = R6/2 & blue = NT. **e** UMAP plots of the human snRNAseq results showing color-coded by cell type (Left), condition (Right).

R6/2 mice are a rapidly progressing transgenic model that express mHTT exon 1 and have features in common with human symptomatic HD, including transcriptional changes^31^. To uncover progressive, cell type-specific, and region-specific transcriptional changes, snRNAseq was conducted on three striatal and cortical samples each from R6/2 and non-transgenic (NT) mice at 8w and 12w of age (Fig. 1a and Supplementary Fig. 1a see “Methods”). snRNAseq data were also generated and analyzed from human HD and control brains (Fig. 1a, e and Supplementary Fig. 1b, c described below). Unsupervised clustering identified 13 clusters in the 8w and 12w striatal samples, and 18 and 16 clusters in the 8w and 12w cortical samples, respectively (Fig. 1b and Supplementary Fig. 1a), which we annotated using known cell type gene markers (Supplementary Fig. 1d). R6/2 and NT cells clearly separate in some of the clusters, such as the 12w D1 + MSNs, which corresponded to a large number of DEGs between the two conditions (Fig. 1b–d and Supplementary Data 1). We also identified DEGs in the excitatory (Ex) and inhibitory (Inhib) neurons, astrocyte (Astro), OLs, and OPC clusters (Fig. 1c). Minimal to no changes were seen in the microglia (MG), vascular cells, and cholinergic neurons (Fig. 1c). These clusters had the smallest number of cells and therefore could lack the power required to identify statistical differences. Regional differences are reflected in the number of cell type-specific DEGs across both regions (Fig. 1c). When we combined all data from both ages and regions, we found no clustering differences for each cell type between age and region, except for cell types that were specific to either the striatum or cortex, e.g., MSNs in the striatum (Supplementary Fig. 1e). The only differences between the age groups were seen as a slight shift within cluster between the 8w and 12w OLs.

**Fig. 2:**
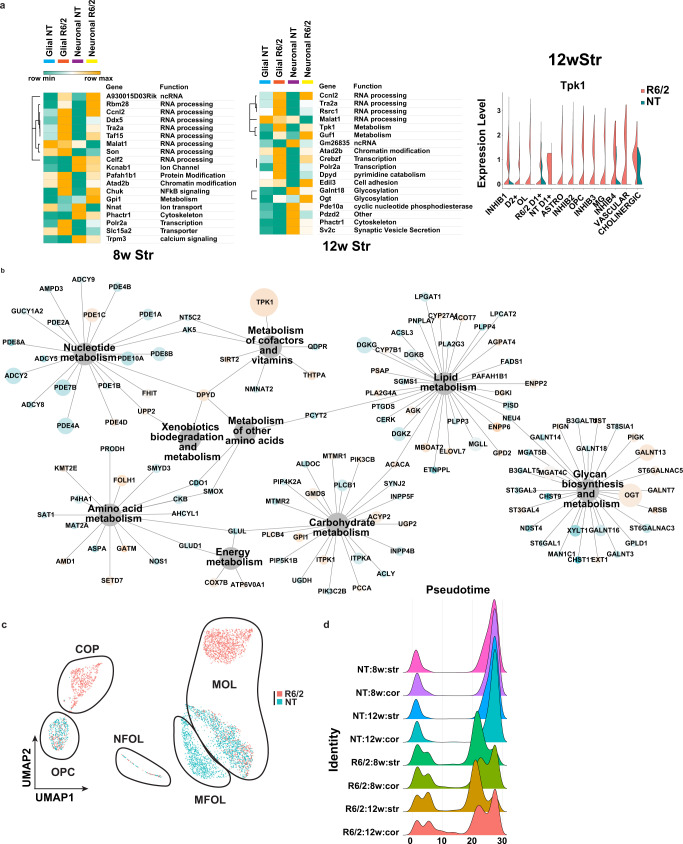
Analysis of differentially expressed genes in R6/2 mice and subclustered analysis of OPCs and OL. **a** Left: Heatmaps and hierarchical clustering of normalized mean expression values in all glial or neuronal cells of the top cell type-agnostic DEGs. Cell color represents row min (seafoam green) and max (orange). Color bars denote NT glial cells (light blue), R6/2 glial cells (orange), NT neural cells (purple), and R6/2 neuronal cells (yellow). RNA processing and splicing (*Ccnl2, Tra2a, ddx5, Celf2,* and *Taf15*) and metabolism (*Guf1, Tpk1*, and *Gpi1*) related genes. Glucose super metabolism pathway genes that include glycolysis, the hexosamine biosynthetic pathway, polyol pathway, and diacylglycerol pathways, include *Ogt, Tpk1, Gpi1*, and *Galant18*. 8w and 12w Str data shown, cortical data in Supplementary Fig. 3a. Right: violin plot of exemplary gene *Tpk1* that show global upregulation in R6/2 mice, across all cell types, from 12w Str. **b** Network showing all KEGG metabolic genes significantly dysregulated across the 12w Str DEGs from every cell type. 12w Str data shown, 8w Str and cortical data in Supplementary Fig. 3b. Node size is equal to the number of cell types in which the gene is found to be significantly dysregulated, and nodes are colored by up and downregulation (orange = up and blue = down). **c** UMAPs of subclustered OPCs and OL in the 12w striatum, colored by genotype. Cluster composition: NT cells are mainly MOLs and MFOLs, or OPCs; while R6/2 cells are COP, NFOL, and MOL. Statistical contrasts: R6/2 vs NT for each cluster, cluster comparisons between R6/2 and NT MOLs, NT MFOLs and R6/2 MOL, COP vs OPCs. 8wStr and cortical data show in Supplementary Fig. 3c. **d** Density plots of cell numbers across pseudotime cell stages, colored by genotype and age.

Gene ontology (GO^36^) enrichment and KEGG pathway analyses were used to investigate the biological implications of the cell type-specific DEGs. The top 10 significant terms revealed that the majority of DEGs, regardless of cell type, are involved in neuronal related functions, including neurogenesis, synaptic function, and glutamate-related signaling (Supplementary Fig. 2a), but certain cell types were enriched for unique terms such as RNA processing in inhibitory neurons. We also identified common terms related to “developmental process” in the cell types with the most DEGs, such as in OLs and OPCs. Similar to the GO analysis data there were recurring KEGG pathways across regions, ages, and cell types as well as sets of unique pathways that group together (Supplementary Fig. 2b). We also identified cell type-agnostic DEGs that were common to both glia and neurons. Figure 2a and Supplementary Fig. 3a show the top multi-cluster DEGs identified in at least 50% of the cell types/clusters per tissue region and age. Many DEGs across both glia and neurons are involved in RNA processing and splicing, and metabolism. KEGG pathway analysis also highlighted glucose metabolic pathways, many of which appeared in the earlier 8w samples (Supplementary Fig. 2b). The dysregulated metabolic genes were found to be in or downstream of the glucose super metabolism pathway that includes glycolysis, the hexosamine biosynthetic, polyol, and diacylglycerol pathways, including *Tpk1* that happened to be dysregulated across the most cell types in the 12w striatum (Fig. 2a). Moreover, *Tpk1* was also among the top dysregulated genes in the 12w cortex, and another glycolytic gene, glucose-6-phosphate isomerase 1 (*Gpi1*), was one of the top multi-cluster DEGs in both 8w striatum and cortex (Fig. 2a and Supplementary Fig. 3a). Both metabolic genes are upregulated in R6/2. We investigated whether there was an enrichment for KEGG metabolic genes in the DEGs and which metabolic pathways were most impacted Fig. 2b, 12w striatum and Supplementary Fig. 3b. *Tpk1, Ogt, Dgkx genes, and Galnt13*, found in sub-pathways related to glucose and lipid metabolism, are among the most commonly dysregulated genes in all cell types.

Taken together, these data indicate that developmental genes, RNA processing, and glucose and lipid metabolism may be core, cell type-agnostic signatures, while cell type-specific changes are more related to cell functions and identity. Further, OPCs and OLs show massive transcriptional dysregulation that includes both developmental genes and functional genes such as MBP.

### R6/2 OPCs are committed to maturation while OLs appear transcriptionally less mature than NT OLs

Given the changes in developmental genes in OPC and OLs, and the unique embedding of R6/2 cells right between the OPC and OL clusters (Fig. 1), we investigated whether these cells might represent intermediate maturation cell states between OPCs and OLs. The OL-OPC data were subclustered, revealing six clusters in the 12w striatum and five clusters in the 8w striatum, 8w cortex, and 12w cortex. Each cluster represented distinct populations of OPCs or OLs comprised of R6/2 and/or NT (Fig. 2c (12wk striatum), and Supplementary Fig. 3c–e, integrated data cross regions, and ages are described in supplementary results and Supplementary Data 2). These subclustered data were then further annotated based on the gene expression markers and annotations defined by Marques and Zeisel et al.^37^ as OPCs, committed oligodendrocyte precursors (COP), newly formed oligodendrocytes (NFOL), myelin-forming oligodendrocytes (MFOL), or mature oligodendrocytes (MOL) (Fig. 2c and Supplementary Fig. 3c). The relative proportions of R6/2 and NT cells in each cell stage and DEG analyses revealed that R6/2 OPCs (OPC & COP) and OLs (NFOL, MFOL, and MOL), at both ages and in both anatomic regions have changes in expression that suggest developmental/maturation impairments. DEGs included: *Mog, Mag, Mbp, Opalin*, microtubule genes, and genes involved in OL maturation, function, and myelination (Supplementary Data 1 and Supplementary Fig. 3e). DEGs involved in glucose and lipid metabolism were also found in OPCs and OLs, including upregulation of *Tpk1*. Pseudotime analysis^38^ further suggested that most R6/2 cells were in transitional cell states between OPCs (pseudotime 0) and MOLs (pseudotime 30+), with many HD cells found in the COP cluster and a cluster of NFOL, while NT cells were mostly either OPCs, MFOL, or MOLs (Fig. 2c, d and Supplementary Fig. 3c–f, these results are further described in the Supplementary Results).

The downregulation of mature OL genes in the R6/2 cells and the distribution of R6/2 cells in intermediate stages of OL differentiation suggests states of abnormal maturation (Fig. 2d) and implies that OPC maturation and subsequent OL differentiation is impaired in R6/2 mice.

### Causal network modeling (CNM) identifies disrupted gene expression networks in R6/2 mice and reveals potential cell type-specific mechanisms of transcriptional change

**Fig. 3:**
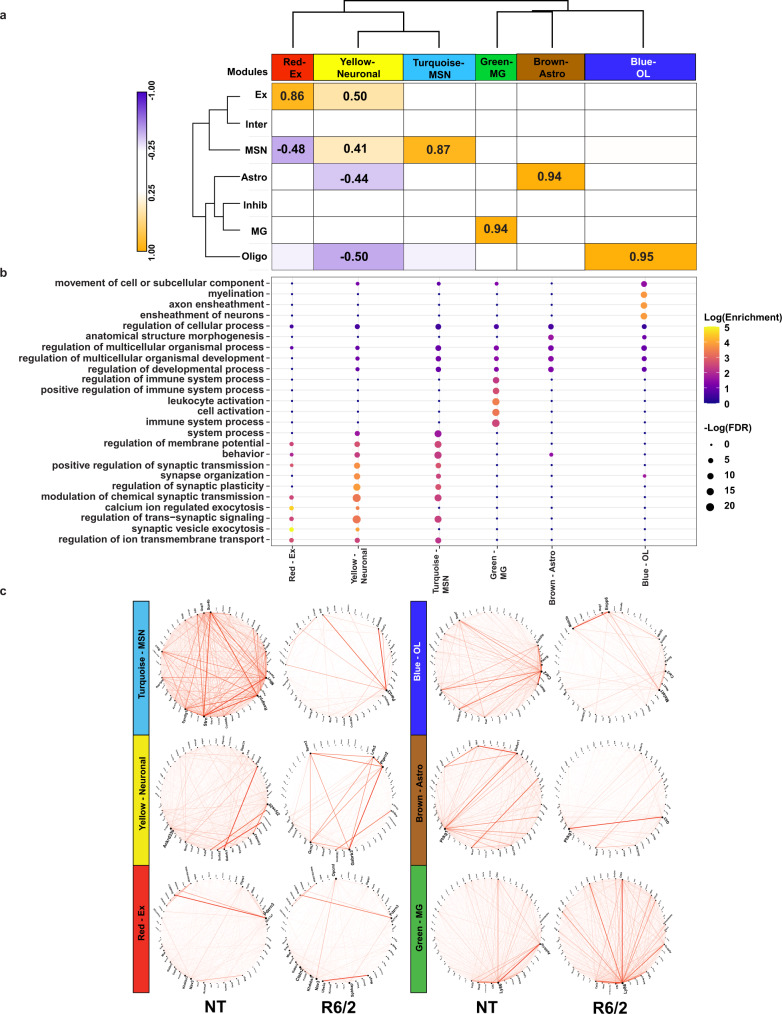
WGCNA analysis of R6/2 mouse snRNAseq data shows cell type-specific changes in network structure. **a** Dendrogram and correlation heatmap showing cell type-specific co-expression modules. Heatmap shows modules highly correlated with each cell type, dendrogram shows clustering of neuronal module together and glial together. Cell color represents column min (blue) and max (orange). Any statistically significant trait-module correlations are shown with correlation value. *P*-values (Supplementary Data 3) are Student asymptotic *p*-values. **b** Top five GO terms per module, showing cell type-specific functional relevance. **c** Circos plots of the top 50 genes with highest kME in NT mice (left) and R6/2 (right). Red lines show connectivity between the top 50 genes. Structural differences can be seen between NT and R6/2.

To investigate disruptions in cell type-specific gene networks in HD, and identify potential key driver genes, we utilized weighted gene co-expression network analysis (WGCNA^39^) and Bayesian causal network modeling (Fig. 1a) to identify causal relationships between genes identified as cell type-specific DEGs and genes correlated within WGCNA network modules^40–42^. After feature selection (“Methods”), we used WGCNA and ran a signed network analysis using cells from all NT samples; 6 gene co-expression modules were detected across cortical and striatal tissues at both ages (Fig. 3a, Supplementary Data 3, and Supplementary Fig. 4). Trait-module correlation analyses showed that our modules were correlated to specific cell types (Fig. 3a). The yellow module positively correlated with neuronal cell types and negatively correlated with glia, and the red, turquoise, green, brown, and blue modules positively correlated with Ex, MSNs, MG, Astros, and OLs, respectively. GO enrichment analysis of gene module members showed enrichment for terms related to each cell type (Fig. 3b). For example, the OL-correlated blue module was enriched for myelination-related terms. Except for the green module, each module was significantly enriched for DEGs determined using the hypergeometric test (Supplementary Fig. 5a and Supplementary Data 3), suggesting that these gene networks are relevant to the disease state and become impacted as the disease progresses. The connectivity of the top module members rank-ordered by eigengene-based connectivity (kME) revealed significant alterations (Fig. 3c).

**Fig. 4:**
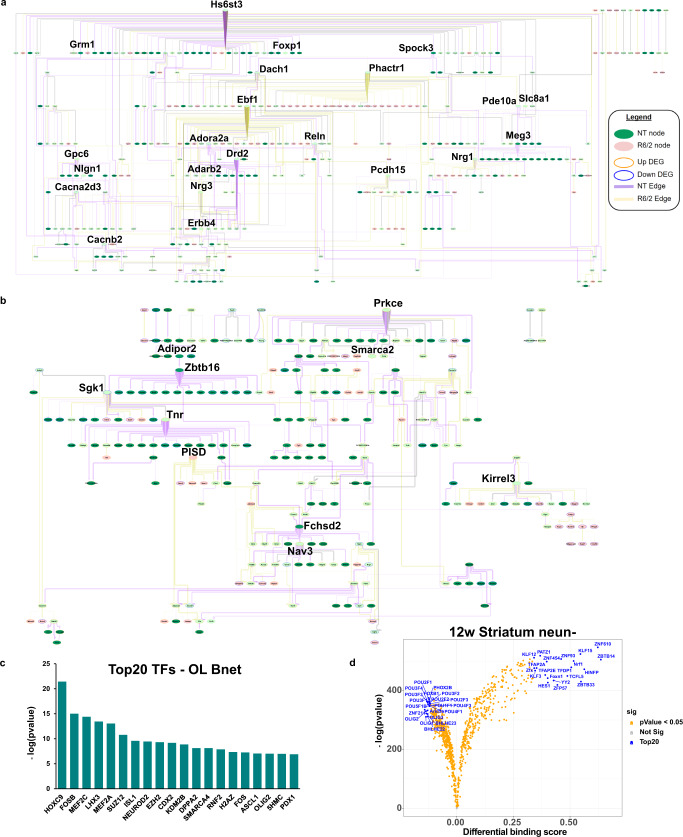
Causal network analysis and ATACseq of glia reveals Prkce, Olig1/2, Sox9/10, and glucose and lipid metabolism as important regulators. **a** MSN bnet. **b** OL bnet. **a**, **b** Both causal networks are merged from NT and R6/2. If a node and edge existed in both the NT and R6/2 bnets, the NT data (edge weight) were used for plotting. Each bnets shows nodes that exist only in NT or R6/2 and nodes that exist in both, as well as new edges and edges retained in the R6/2 data. Each bnet was also plotted using a hierarchical structure to show the direction of causal flow. In each plot, genes with a high degree of outward centrality (>10 outward edges) are highlighted by increased gene name size, as well as genes that connect between two genes that have a high degree of outward centrality. We consider these highlighted genes key drivers of the network. Color scheme is as follows: Edge (purple = NT, yellow = R6/2, gray = both), node fill color (green = NT node, pink = R6/2 node, light green = both), node outline color (orange = upregulated, blue = downregulated). MG, Astro, and Ex neuron bnets are in Supplementary Fig. 5b–d. **c** LISA analysis of OL causal network gene members, showing the top 20 regulatory transcription factors. **d** Volcano plot showing differential binding scores, and −log(*p* value) differences of TF binding in open chromatin in 12w NeuN- striatal cells. blue = top20 by differential binding score, orange = *p* value < 0.05. 8wStr, cortical, and all NeuN+ data can be found in Supplementary Fig. 6b.

To understand the potential cell type-specific causal connections between these genes we applied a Bayesian causal network modeling approach (see “Methods”), using the cell x gene data matrix which was filtered and run for each individual cell type and used for network structure learning. Gene features were selected as input by using only that cell type’s DEGs and gene members from the correlated WGCNA module (Fig. 4a, b, Supplementary Fig. 5b–d, and Supplementary Data 4). We explored the MSN and OPC/OL bayes nets (bnets) in detail for two reasons: (1) since MSN are the most studied cell type in HD the bnet should recapitulate previous findings and also reveal both known and undefined interactions between known dysregulated genes, providing validation for our approach, and (2) both cell types were the most impacted in our mouse model (total number of DEG) with the OPCs and OLs showing the largest number of DEGs that suggested developmental deficits. The merged NT and R6/2 bnets per each cell type are shown in Fig. 4a, b. We enlarged genes that represent key drivers (hub genes with high outward centrality, or genes connecting 2 hubs) which are potentially causal regulators of downstream nodes.

The MSN bnet includes genes involved in MSN development/identity, function, and genes implicated in HD, including *Ebf1*, a key driver identified in the R6/2 bnet (yellow edges) and involved in striato-nigral MSN development^43,44^. Genes of the indirect pathway in D2 + MSNs, including *Adora2a, Drd2, and Penk*, were all downregulated and only show NT causal interactions (purple edges), indicating a loss of function of these genes^45^. Furthermore, *Drd2* is a parent node of *Penk*, which is not only a downstream target of *Drd2* signaling and dysregulated in HD^14^, but is transcriptionally regulated by *Drd2* expression through dopamine-induced activation^46^, thus validating the approach.

We next explored the OPC/OL bnet (Fig. 4b) and found *Prkce, Sgk1, Zbtb16* and *Tnr* as key drivers. Prkce is functionally regulated by DAG and transcriptionally by Zbtb16^47^, a zinc finger binding protein that is involved on OL maturation and myelination, is found downstream of Adipor2, an adiponectin receptor that regulates glucose and lipid metabolism. Downstream of *Zbtb16* is serum- and glucocorticoid-inducible kinase 1 (*Sgk1)*, which is normally upregulated in OLs during cellular stress and regulates many ion channels and solute carrier proteins involved in metabolic pathways and glucose uptake (e.g., ref. 48), such as GLUT1, GLUT4, and glutamate transporters. *Sgk1* is downregulated in R6/2 mice indicating a potential loss of function in HD—see supplementary results for additional validation studies. Exploration of downstream nodes reveals a connection between *Smarca2*, which is a protein in the SWI/SNF family involved in gene expression and chromatin remodeling in OLs, and *Prkce*. Smarca2 (BRM) and Smarca4 (BRG1) play roles in OPC and OL development, including promoting OPC differentiation^49,50^. The majority of the outward edges from key drivers are NT specific, indicating a loss of causal connection to downstream nodes in the R6/2 mice. Transcription regulator analysis of the network gene members using LISA^51^ revealed the network is enriched for targets of Smarca4, and Olig2, as well as other regulators previously highlighted for HD, including Suz12, Fos, and Mefc2 (Fig. 4c and Supplementary Data 4).

Finally, to complement these findings using a completely different knowledge-driven approach, and provide additional interpretability, we also included cell type-specific gene regulon network analysis using IRIS3^52^. This analysis identifies predicted transcriptional regulators from prior knowledge which we then used to overlay onto our data driven causal network (Supplementary Fig. 5e, Supplementary Data 4). We found a significant enrichment of genes and regulons already within our causal network (*p* < 2.373e−201, exact hypergeometric probability), and many connections between several of these regulons, including OLIG2, and our key drivers. ELF5 and E2F3, which were two regulons predicted from the IRIS3 analysis, both regulate *Prkce* and have previously been implicated in OPC maturation and HD transcriptional dysregulation^5,53^. These findings further validate the causal networks and together these data suggest an interconnected process between OPC/OL development and lipid and glucose metabolism and known HD-related genes.

MG, Astro, and Ex neuron bnets are described in the supplementary results.

### ATACseq of glial-enriched nuclei identifies regulators underlying transcriptional pathology in HD glia

To understand the drivers of gene expression changes in non-neuronal cells (e.g., glia) versus neurons, and validate the transcriptional regulator analyses (LISA & IRIS3), we performed ATACseq on NeuN+ and NeuN- sorted nuclei from both the striatum and cortex of the same R6/2 mouse cohort (Supplementary Fig. 6a and Supplementary Data 5). The neuronal protein NeuN is localized in the nuclei and perinuclear cytoplasm of most neurons. We performed footprinting analysis using the ATACseq data and TOBIAS^54^ which revealed developmental changes in the glia-enriched NeuN- data (Fig. 4d (12w striatum) and Supplementary Fig. 6b, and Supplementary Data 5), and enrichment for immediate early genes in the neuron-enriched NeuN+ data. Among the top 20 TFs in the NeuN- data that showed differential binding between R6/2 and NT, we found Sox9 and 10 were significantly decreased in the 8wk striatal data, and *Olig1* and *Olig2* decreased in the 12wk striatal data. Interestingly, when all the samples were grouped and we compared the top 20 up and down TFs per an age and region, there were some overlapping TFs between the 12w cortical and both striatal samples, but these were in opposite directions (e.g., *Hes1* and *Zbtb14*, Supplementary Fig. 6b, c). The 8w cortical samples had the least similarities compared to all other regions and ages (Supplementary Fig. 6b, c) and showed a number of HOX genes within the top 20 TFs with reduced binding. The cortical data showed differential binding of other known HD genes such as Egr1 and Sp1.

Since we did not have ATACseq data from our human samples we next conducted global chromatin accessibility prediction using our human snRNAseq data and the BIRD tool^55^. BIRD analysis revealed differential peaks at genes important for OPC/OL maturation, such as *SMARCA4*, which were dysregulated in the snRNAseq data in both mouse and human samples (Supplementary Fig. 6a).

Independently, both analyses of ATACseq data suggest maturation impairments in HD OPC/OLs. Together with the snRNAseq data, a coordinated network of regulators and downstream effectors that implicate known OL developmental genes (e.g., *SMARCA4* and *Olig2*), and other potential regulators (e.g., *PRKCE*) is observed.

### Single nucleus RNAseq from HD and control cingulate, caudate, and nucleus accumbens identifies several heterogeneous OL lineage cells and altered maturation states

Given the altered gene expression in OL lineage cells in R6/2 mice, we investigated whether mHTT expression also impacted gene expression in OPCs and OLs in human HD post-mortem tissue. snRNAseq was carried out on 66 samples from 29 donors (3 grade I, 4 grade II, 4 grade III, 3 grade IV, 5 juvenile-onset HD, and 10 matched controls—the demographics of whom are outlined in Supplementary Data 6). To define the pathology in different brain regions, we microdissected the cingulate cortex, the caudate, and the nucleus accumbens from frozen brain tissue as outlined in Fig. 1a. All major lineages were identified in the 290525 nuclei analyzed (Supplementary Fig. 1e). Projection of nuclei in UMAP space shows that nuclei of the same lineages largely occupy neighboring space (Fig. 1e and Supplementary Fig. 1d, e (tSNE)), without distinct donor or batch related colocalization after correcting for batch effects (Supplementary Fig. 7a, b). We detected changes in gene expression in all cell types; for this study, we focused on cells of the OL lineage.

**Fig. 5:**
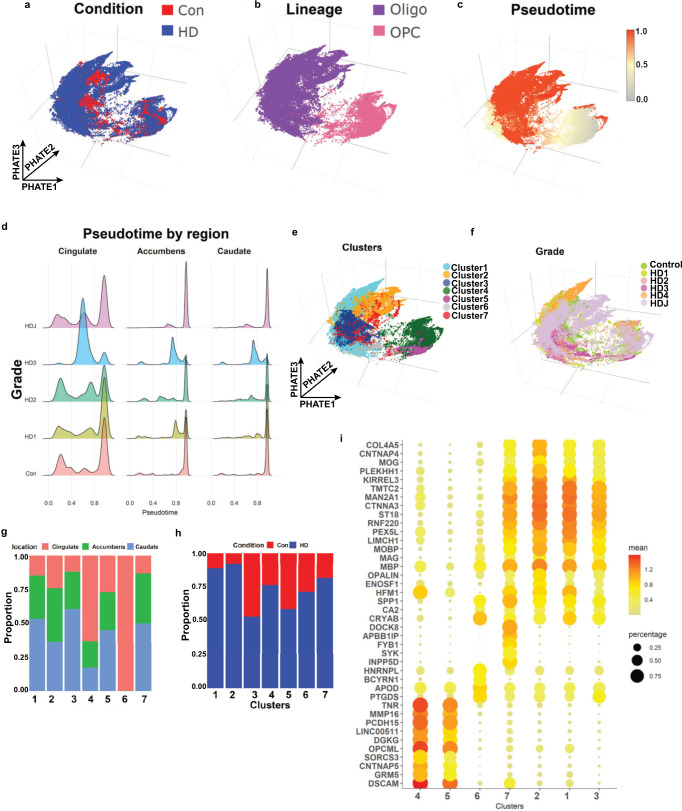
Huntington disease oligodendrocytes are less mature. Projection of control and HD nuclei in the PHATE dimension color-coded by condition (**a**), lineage (**b**), pseudotime value (**c**), cluster (using the Levine algorithm) (**e**), and HD grade (**f**). Note that OPCs have the lowest pseudotime values in **c**, as OPCs were set as root nodes, while OLs had high values. **d** Pseudotime values are shown in histograms across brain region and HD grade. Note that the proportion of nuclei with intermediate pseudotime values is higher in HD, especially grade III. The relative contribution of anatomic region (**g**) and condition (**h**) to each cluster is shown in bar plots. **i** Gene expression dot plots showing normalized expression of select cluster marker genes, with color denoting expression levels and circle size denoting the proportion of nuclei expressing the gene of interest.

We focused on OLs and OPCs (Fig. 5a, b) and analyzed 80199 OL and 13844 OPC nuclei in isolation from other lineages. Projecting OL and OPC in their own reduced dimension space (PHATE reduction—see “Methods”) shows a continuous trajectory from OPCs to OLs, and separation between HD and control nuclei (Fig. 5a, b). To examine the differentiation states of these cells, using well-established methods^56^, we calculated the relative ordering of cells along a pseudotime dimension (Fig. 5c). Similar to our mouse data, examination of pseudotime values per anatomic region in control, grades I–III HD, and Juvenile onset HD nuclei show altered maturation states across brain regions and grade in HD. That is, across all brain regions examined, HD nuclei showed a relatively larger proportion of cells with intermediate pseudotime values compared with controls, which is more pronounced with increasing HD grade, particularly in HD grade 3. Conversely, in juvenile onset HD (HDJ), the effect was less appreciable in the cingulate cortex, and more pronounced in the striatum, with the majority of caudate and accumbens OPCs showing intermediate pseudotime values (Fig. 5d). The results suggest that HD maturation pathology is at least partially progressive with HD grade, and that HDJ maturation pathology affects mainly OPCs.

We next performed unbiased sub-clustering of OL and OPC nuclei and identified 7 sub-clusters (Fig. 5e). Most subclusters contained a mix of cells from all three regions (Fig. 5f) and HD grades (Fig. 5g, h), although in clusters 4 and 6 most nuclei were derived from the cingulate, and in clusters 1, 3, and 7 caudate nuclei represented the largest proportion (Fig. 5g). Most clusters contained mixtures of nuclei from both HD and controls, but a number showed a preponderance of one or the other (Fig. 5h) with the caveat that our dataset harbored relatively larger numbers of HD nuclei versus control (Con 17955, HD 76088). With that caveat, Cluster 2 was mostly composed of HDJ nuclei, while cluster 6 was composed of a preponderance of HD3 nuclei (Supplementary Fig. 7c). Examination of select gene markers shows that clusters 4 and 5 represent OPCs with relatively high expression of OPC markers *TNR* and *DSCAM* (Fig. 5i and Supplementary Fig. 7d) and low expression of gene markers for mature OLs. Compared to cluster 5, cluster 4 shows lower expression of OPC genes *BCAN*, *VCAN*, *PDGFRA*, and *CSPG4*, but a higher proportion of cells with *TCF7L2* expression, suggesting this cluster represents differentiation committed OPCs^57^ (Supplementary Fig 7d). Conversely, clusters 1, 2, 3, and 7 show relatively high expression of OL genes *CNP, PLP1*, and *MBP* (Fig. 5i). Among the former, cluster 2 shows the highest expression levels of *OPALIN* and *MOG*, suggesting it is most mature (myelinating). Cluster 7 showed expression of both OL genes (although at comparatively lower levels) and the OPC gene *DSCAM* and is interpreted as an intermediate state between OL and OPC lineages. Likewise, cluster 6 showed expression of the immature OL gene *CA2* as well as other OL genes including *APOD, PTGDS*, and *CRYAB*, but not myelin genes. It is thus also interpreted as immature OL. Interestingly, the HD-enriched clusters 1, 2, and 7 showed higher expression levels of *KIRREL3* compared with the control-enriched cluster 3. *KIRREL3* is a gene shown to be highly expressed in OL residing in chronic inactive lesions of multiple sclerosis^57^, and was identified as a key driver in our causal network model. The cluster markers are provided in Supplementary Data 7.

These data show a progressive loss of OL maturation in HD that spans different brain regions. but appears more pronounced in the striatum. The data also suggests similar mechanisms between the mouse and human HD samples, showing differential expression of previously described key drivers, but also unique features of human HD OLs with immune-related genes changes.

### Differential gene expression analysis reveals further differences between HD and control OLs

**Fig. 6:**
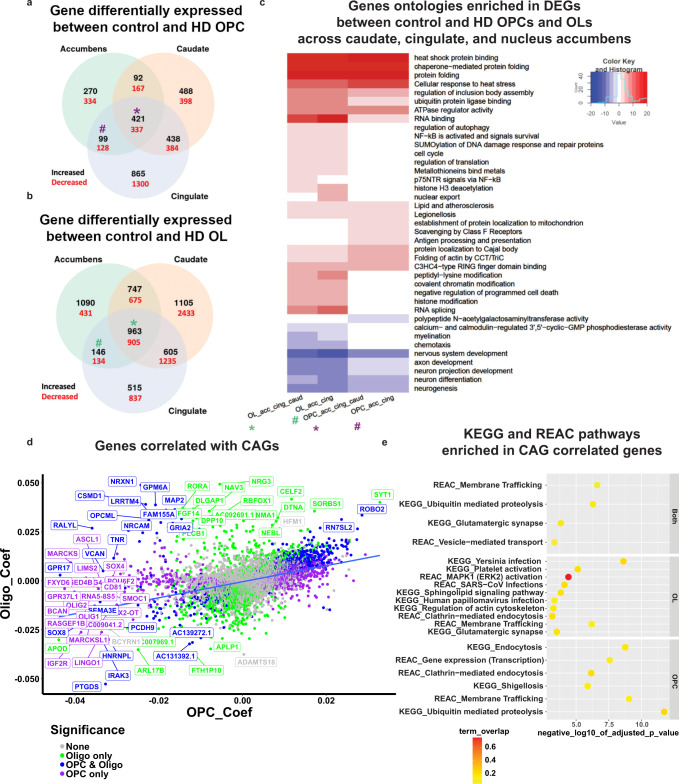
Differential gene expression analysis of HD and control OPCs and OLs. Venn diagram analysis of the DEGs in OPCs (**a**) and OLs (**b**). The number of DEGs that are increased (black) or decreased (red) in HD nuclei is highlighted per overlap sector. The stars indicate the DEGs that are shared across all regions, and the # indicates the DEGs shared between the Cingulate and Accumbens. **c** Gene ontology (GO) term analysis of differentially expressed genes in select sectors of the Venn diagrams HD versus control OLs and OPC (from panels **a**, **b**). The * and # signs correspond to the DEGs shared across all regions and DEGs shared between accumbens and cingulate OL and OPCs, respectively (purple = OPC DEGs, and green = OL DEGs). The sign of the negative log10 of the adjusted p value indicates the direction of changes; positive sign corresponds to genes increased in HD, and negative sign corresponds to genes decreased in HD. Heat shock protein encoding genes HSPA1A, HSPH1, HSPA4L, HSP90AA1, HSPB1, HSPA4, HSPD1, HSPA1A, HSPA1B, and HSPB1. **d** Scatter plot of the correlation coefficients of genes that correlate with CAG repeats in OPCs (*y*-axis) and OLs (*x*-axis). The graph plots the regression coefficients of each gene in OLs versus OPCs; the upper right quadrant represents genes with positive correlations in both OPCs and OL, the lower left quadrant genes that have negative correlations in both. The color of the genes correspond to whether the coefficient was significant in OLs only (green), OPCs and OLs (blue), or OPCs only (purple). **e** KEGG and Reactome pathway enrichment analysis of the genes that significantly correlate with CAG repeats in OPCs and OLs (top panel), OLs (middle panel), or OPCs (lower panel). The negative log10 of the adjusted *p* value is indicated on the *x*-axis, and the pathways on the *y*-axis. The color of each circle corresponds to the percentage of overlap between the CAG-correlated genes and the genes in each pathway.

We next identified significant DEGs between HD and control OL and OPC nuclei in different regions; the number of significant DEGs unique to and shared by respective anatomic regions is shown in Venn diagrams for OLs (Fig. 6a and Supplementary Data 8) and OPCs (Fig. 6b and Supplementary Data 8). Given that the neurodegeneration is detected in the caudate nucleus at the earliest stages of HD and that pathology in the nucleus accumbens and cortex is typically seen in more advanced disease, we reasoned that comparing DEGs in these regions is informative in the following ways: (1) DEGs that are shared among the caudate, accumbens, and cingulate likely represent pervasive or core transcriptional pathology in different anatomic regions regardless of disease severity. (2) DEGs shared between the relatively preserved nucleus accumbens and less severely affected cingulate cortex likely represent early pathologic alterations that may be compensatory in early stages of the disease and are lost in more advanced stages. This does not preclude the possibility that any number of these DEGs may represent cell-autonomous changes due to mHTT in OL and OPCs.

With this insight, examination of significant DEGs in these regions highlights a number of themes; first, myelin related and OL identity genes (*e.g.,MAG*, *MBP*, *MOBP*, *MOG*, *OPALIN*, *PLP1*, *CNP*, and *OLIG1* and *OLIG2*) were downregulated in OLs of all areas in HD, (Supplementary Data 8). This was reflected in a negative enrichment of the GO myelination in HD OL’s across all three brain regions (Fig. 6c). Second, multiple heat shock encoding and heat shock response genes were increased across all anatomic regions, suggesting widespread, pervasive pathology in HD OLs (Supplementary Data 8). Multiple metallothionein genes (e.g., *MT2A*, *MT3, MT1X*, *MT1M*, and *MT1E*) were also increased in all brain regions in HD (Supplementary Data 8). *SPP1*, which encodes a secreted protein that is increased in demyelination and remyelination^58^, was also increased in all these regions. *CA2*, a gene encoding a carbonic anhydrase enzyme expressed in immature OL and mature OLs but not OPCs^59^, was increased in cingulate OLs (validated in Supplementary Fig. 8b–e).

To determine whether similar metabolic genes were dysregulated in our human OPC and OLs that were found in our mouse data, we overlapped human OPC and OL DEGs with the dysregulated metabolic genes in the 12w striatum data and found a large overlap with these DEGs (Supplementary Fig. 8a) including *DGKx*, *GALNTx* genes, *PTGDS*, and *TPK1*. Furthermore, several DEGs shared between the accumbens and cingulate OLs were related to metabolism, including adipogenesis (*ARL4A*, *COQ3*, *CHUK*, *ABCA1*, *GBE1*, and *ME1*—increased in HD OLs), fatty acid metabolism (*EVOVL2* and *PLA2G6*—decreased in HD OLs), and pyruvate metabolism (pyruvate kinase M1/M2 *PKM* - decreased in HD OLs). These results implicate metabolic pathways, including lipid and glucose metabolism in HD pathology (Fig. 6c and Supplementary Data 8). The involvement of immune genes we observed in HD-enriched clusters is also reflected in the enrichment of immune-related ontologies in the HD OLs DEGs, including NFKB activation and inflammasome (Fig. 6c and Supplementary Data 8). Similar to the mouse data, we also see terms related to nervous system development, ion channels, and cell adhesion (Fig. 2a and Supplementary Data 8).

These results confirmed the pseudotime analysis showing a decrease in OL maturation in HD. Downregulation of OL-specific functional genes and a significant enrichment of metabolic genes, similarly identified in our mouse data, suggest a common theme and possible connection between metabolic processes and OL development in HD.

### Dysregulated gene expression is related to numbers of CAG repeats

The length of CAG repeats varied among our donors, and even between regions in the same donor (Supplementary Data 6). To determine if any of the OL or OPC genes varied as a function of the numbers of CAG repeats, we conducted a regression analysis with gene expression as response variable and CAG repeats as explanatory variable. We collapsed cells from each sample and used the pseudobulk samples as input for the regression analysis, corrected for batch and brain region, and only extracted the significant CAG coefficients (Supplementary Data 7). A number of genes showed significant correlations between expression and CAG repeat lengths, some in OPCs or OLs or both (Fig. 6d). Among genes with negative correlations in OPCs are transcription factors *OLIG1* and *OLIG2*, *ASCL1*, *SOX2,* and *SOX4*, which play roles in OL-lineage development, along with *IGF2R*, suggesting that progression through the OL lineage is further inhibited with longer repeat length. Indeed, OPC lineage genes including *OPCML* and *CSPG4* were negatively correlated with CAG repeat length (Fig. 6d). Moreover, *PTGDS*, a cluster 6 marker, had the most negative coefficients in both OPCs and OLs as a function of CAG repeat length, implicating prostaglandin synthesis in the severity of HD pathology. Some of these genes also were identified in our OL bnet as key drivers, including: *SGK1*, *TNR*, and *NAV3* (Fig. 4b). We also investigated KEGG and REAC pathways that were enriched in genes correlated with CAG repeat lengths (Fig. 6e and Supplementary Data 7). Among the pathways that are enriched in OLs with increasing repeat lengths are those of inflammation, which is more pronounced in human brain than in the mice, sphingolipid signaling, and ERK2 activation, known to control myelination^60^. Both OLs and OPCs show enrichment in genes related to glutamatergic synapses and ubiquitin-mediated proteolysis. When we examined the OL genes with negative coefficients, we found that a number of them are involved in cholesterol metabolism including (*DHCR7*, *DHCR24, ABCA2*, and *ACAT2* – Supplementary Data 7), which further implicates lipid metabolism as central to OL pathology in HD.

### Validation of OL pathology in human HD and mouse data

**Fig. 7:**
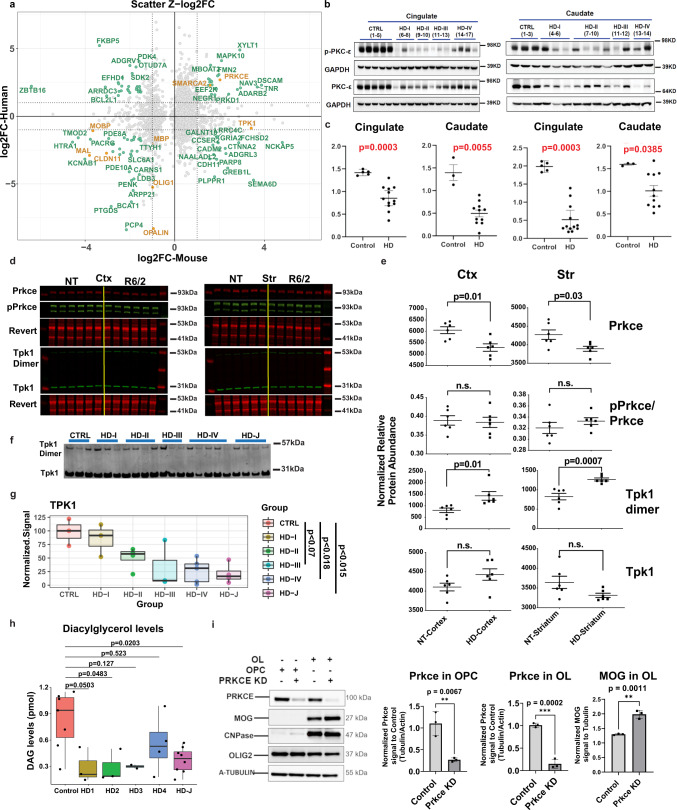
Western, lipidomics, and cellular analyses validates HD differences in TPK1 and PRKCE. **a** Scatterplots of Z-score log2 fold change values comparing mouse and human data in 12w striatum versus human caudate OL DEGs. Genes with |Z-log2FC| values > 1 are highlighted in seafoam green and OL maturation genes are highlighted in orange, showing concordance between species for PRKCE and OL maturation genes, and discordance of TPK1 expression. **b** Western blot of PRKCE and phospho-PRKCE in HD and control patient cingulate cortex and caudate. **c** Quantification of western blot results. Two-tailed Mann Whitney test used for each statistical analysis. Exact *p*-values: Cingulate: PKCE-0.0003, p-PKCE-0.0003; Caudate: PKCE-0.0055, p-PKCE- 0.0385. *n* = 3 control and 11–12 HD caudate samples, and 5 control and 11–12 HD cingulate samples. Data shown as mean +/− SEM as error bars. **d** Licor images of Prkce, pPrkce, TPK1, and respective revert in R6/2 and NT striatum and cortex. **e** Quantification of licor results. One-way ANOVA used for statistical analysis. *n* = 6 NT and 6 R6/2, biologically independent samples. Data shown as mean +/− SEM as error bars. **f** Western blot of TPK1 in human caudate samples from juvenile HD, HD grades 1–4, and control patients. **g** Quantification of human TPK1. Statistical analysis was done using a one-way ANOVA and Tukey HSD posthoc, comparing control to each adult HD grades (adjusted *p* = 0.979, 0.221, 0.070, and 0.018) and control to juvenile HD (*p* = 0.015). Data shown as median (center line), first and third quartile (Inner quartile range, box), and min and max values as whiskers. **h** DAG levels quantified from HD and control patient brains showing significant decreased DAG levels in HD brains. One-way ANOVA and Tukey’s HSD posthoc used for statistical analysis, comparing control to each adult HD grades. *n* = 7 control, 3 HD1, 3 HD2, 2 HD3, 4 HD4, 8 HD-J, biologically independent samples. Data shown as median (center line), inner quartile range (box), and min and max values as whiskers. **i** Western blot of PRKCE, MOG, CNPase, OLIG2, and A-Tubulin in OPC and OLs +/− K/D of PRKCE. Two-tailed Mann Whitney test used for statistical analysis. *n* = 3 biologically independent samples per group. Data shown as mean +/− SEM as error bars. For western blot results, source data are provided as a Source Data file.

**Fig. 8:**
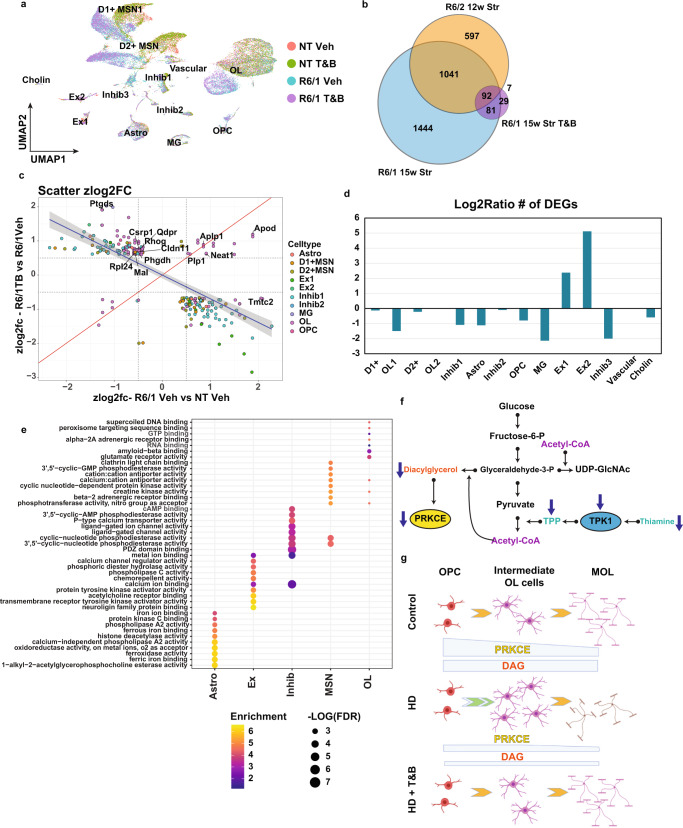
Thiamine and biotin study in R6/1 mice shows rescue of OL maturation DEGs and other cell type DEGs. **a** UMAP showing the R6/1 and NT mouse data colored by genotype and treatment. **b** Venn diagram comparing genotype DEGs in 15w R6/1 mice and 12wStr of R6/2 mice against each other and treatment effect DEGs from R6/1T&B treated versus vehicle. **c** Scatterplot showing Z-score log2FC of all genes overlapping between genotype and treatment effect DEGs. Colored by cell type origin. OL and Inhib1 neurons show the most rescued DEGs. Quadrants 1 and 3 represent rescue of expression and 2 and 4 represent exacerbation. **d** Barplot showing the log2ratio of the number of significant DEGs comparing R61 vehicle versus NT vehicle to R6/1T&B versus NT vehicle. **e** Top 10 GO terms of overlapping DEGs per cell type (R61 vehicle versus NT vehicle to R6/1T&B versus NT vehicle). **f** Illustration of metabolic pathways impacted in HD. **g** Illustration showing how PRKCE and DAG levels regulate OPC commitment to differentiation and MOL maturation in control and HD, and how T&B treatment rescues maturation impairments. Created with BioRender.com.

Many genes that regulate OL maturation or were identified as key regulators were similarly dysregulated in HD patient and mouse data including: *MOBP, MAL, CLDN11, MBP, OLIG1, OPALIN, PRKCE*, and *SMARCA2* (Fig. 7a). We performed WB analysis to confirm dysregulation of key genes *PRKCE* and *TPK1*. Additional investigation and validation of OL genes and other metabolic genes was also conducted (Supplementary results and Fig. 8). Protein levels of PRKCE, and phospho-PRKCE were significantly decreased in the cingulate and caudate of HD brains and the cortex and striatum in the 12w R6/2 mice (Fig. 7b–e). Both species showed an increase in *PRKCE* RNA levels, opposite of the protein data. The ratio of p-PRKCE to PRKCE was not altered though, suggesting that reduction in active PRKCE is related to reduced protein levels (Fig. 7b–e).

Since *TPK1* was found to be dysregulated in both mouse (up) and human (down) data at the RNA level in OLs and OPCs, we assessed the protein levels of the monomer and active dimer form of TPK1. Figure 7f, g shows a decrease of TPK1 (monomer and dimer) in HD patient tissue with HD grades 3 & 4 (At adjusted *p*-value <0.1 for 3, and <0.05 for 4), and in juvenile HD (adjusted *p* < 0.05), consistent with RNA expression data, whereas TPK1 dimer is increased in the R6/2 striatum (Fig. 7d, e). The mouse and human data are discordant from each other which may indicate a loss of function of expression in humans and compensatory increase in the mice or other unknown mechanism. Nonetheless, the data confirms that *TPK1* is dysregulated in both human HD and murine model of HD.

Given the potential contribution of DAG to OL development and as a substrate of PRKCE—a central hub of the OL causal network, we evaluated DAG levels using lipidomic profiling of control brain versus HD in the cingulate. A significant decrease in DAG levels was observed in juvenile HD brain as well as grade 2 HD brains relative to controls (Fig. 7h). These data support the hypothesis that glucose and lipid metabolism, and specifically DAG signaling, potentially through PRKCE, could be playing an important role in the OPC/OL maturation changes we see between HD and control patients. This is further supported by the reduction in TPK1 in HD brains due to the involvement of thiamine in the production of acetyl-CoA, which is then used during DAG formation. Given this finding along with the results demonstrating the reduction of PRKCE in human tissue, together with the causal network analysis placing PRKCE at the top of the OL/OPC network upstream to several maturation genes, we hypothesized that it played an important role in promoting OL differentiation. To test this hypothesis, we knocked down Prkce in primary murine OPC cultures, and differentiated these cells into OLs. The cultures expressed OLIG2, and OLs expressed CNPase. Compared with scrambled siRNA, siRNA specific to *Prkce* effectively knocked down the protein (Fig. 7i). The levels of MOG were significantly increased by Prkce knockdown, supporting that the downregulation of Prkce leads to increased OL differentiation. Thus, the loss of PRKCE—as seen in our western blot data—in both human and mouse HD OPCs/OLs—would lead to increased OPC commitment to differentiation and an increase in COP cells, seen in our snRNAseq data.

These results provide validation of our causal network approach to identify key regulatory genes, and suggests important roles for glucose, thiamine, and lipid metabolism, through DAG and PRKCE, in regulating OL maturation in HD.

### High Dose thiamine and biotin rescues transcriptional dysregulation in neurons and altered OL and OPC developmental genes in a mouse model of HD

Given that both mouse and human data showed alterations in TPK1 and SLC19A2, and these may regulate PRKCE thorough DAG, we hypothesized that altered signaling due to TPP deficiencies may be contributing to gene expression and cell maturation differences. We, therefore, tested whether high doses of thiamine and biotin (T&B) treatment, similar to that used to treat HD-like phenocopy diseases such as biotin-responsive basal ganglia disease^33^, would rescue gene expression changes including OL maturation genes. Furthermore, due to the discordant RNA expression changes in our mouse and human data we speculate that the increase in TPK1 was compensatory in the HD mouse model. Considering TPK1 was only increased at 12w and not 8w, we suspect that these compensatory changes are responding to earlier metabolic changes and tested whether targeting thiamine metabolism at a relatively early timepoint prior to any documented changes in TPK1 expression^61^, would rescue the dys-maturation. For this study, R6/1 mice were used given symptoms are delayed by several weeks relative to R6/2 mice^31^, thus allowing a greater window to observe effects of a given treatment. R6/1 and NT mice (8w-old) were treated with vehicle or T&B for 7wks to a time point at which transcriptional changes are observed, before striatal tissue was collected and analyzed using snRNAseq (Fig. 8a). MSNs, inhibitory neurons, OPCs, OL, and Astros showed the most DEGs between R6/1 and NT vehicle-treated mice (Supplementary Data 10). Comparing R6/2 and R6/1 DEGs for each cell type, we found high correlation between HD models and a significant overlap in DEGs, including between OPC and OL maturation genes (Fig. 8b) supporting the use of R6/1 mice for the supplementation study. When we evaluated DEGs between R6/1T&B treated and vehicle-treated mice (treatment effect), for each cell type, there was a significant overlap of genes impacted by T&B treatment and genotype DEGs (Fig. 8b). Figure 8c shows a scatterplot of the overlapping DEGs between the T&B treatment effect (R6/1 + T&B vs R6/1 + vehicle) and the genotype DEGs (R6/1 vs NT) for each cell type, which shows significant discordance between the genotype DEGs and the treatment DEGs, indicating rescue of these transcriptional alterations. This translated into a decrease in the number of significant DEGs detected for each cell type ((R6/1 + T&B vs NT) compared to (R6/1 + Vehicle vs NT)), except for the Ex neurons which actually had an increase in DEGs (Fig. 8d). Interestingly, the cell types with the most genes rescued by T&B treatment (discordant values) were OL-lineage cells and *Adarb2*+ interneurons that represent inhibitory neuron subcluster 1 (Inhib1 (Fig. 8a)). Based on the reduction of DEGs detected, OL, MSNs, Interneurons, Astros, and OPC all had a large decrease in the number of DEGs detected (115, 176, 378, 129, and 82 DEGs, respectively). Within the OPCs and OLs there was significant rescue of maturation-related DEGs *Clnd11* and *Mal*, and a further increase of *Neat1*, which was increased in caudate-parenchymal human HD OLs, and is upregulated during OL maturation. Several genes that correlated with CAG repeat length, e.g., *Ptgds, Phgdh*, and *Tmtc2*, were rescued by T&B treatment. GO enrichment analysis also revealed the molecular functions of the genotype DEGs that were rescued from T&B treatment (Fig. 8e). In Astrocytes there was a significant rescue of iron metabolism-related genes, Ex neurons showed rescue of neuroligin binding and calcium signaling, and the MSNs showed rescue of cyclic nucleotide phosphodiesterase activity, GABA receptor activity, calcium transport, creatine kinase activity, and electron transport chain genes. Similar to MSNs, the inhibitory neurons showed rescue of calcium-related genes, cyclic phosphodiesterase activity, and creatine kinase activity, but also showed unique terms such as glutamate receptor activity, LDL binding, neurotrophic TRK receptor, and fructose binding. Lastly, the OPCs and OLs showed rescue of glutamate receptor activity, RNA binding, creatine kinase activity, calcium-related genes, and GTP binding.

These results (a) support the hypothesis that common metabolic changes across cell types in HD contribute to driving cell type-specific transcriptional changes and that (b) specifically thiamine metabolism deficits may be contributing to OL maturation deficits.

## Discussion

The studies above describe a systematic and in-depth analysis of single cell transcriptomics of HD mouse models and human patient brains leveraging CNM to implicate key drivers of gene expression pathology. Using snRNAseq, we identified dysregulated genes across multiple cell types and cell type-specific changes that may drive the functional changes seen in each cell type. In addition to specific changes in neurons, such as D1 and D2 MSNs, a large number of changes were seen in the OL lineage that related to development and maturation processes. We defined a progressive dys-maturation phenotype that spans multiple brain regions in both human and mouse HD. CNM identified potential key genes and molecules with putative causal roles in cell type-specific alterations, several of which were connected to metabolic functions, cell maturation, and OL/OPC-identity genes. This includes *PRKCE*, a signaling protein regulated by DAG, which causally interacts with *SMARCA2* and *OLIG2*, both important in OL maturation. Functional studies validated PRKCE’s role in promoting OL maturation. ATACseq data provided further validation demonstrating decreased accessibility for genes regulated by known OL developmental TFs (SOX9 and 10, OLIG1 and 2, and ASCL1)^62^, further implicating abnormalities in OL differentiation in HD. These data provided a framework to build targeted therapeutics, as illustrated by treatment with T&B that restored many of the maturation and transcriptional deficits and provides further validation of our approach.

Recent single nuclei studies identified common and cell type-specific transcriptional alterations in R6/2 and Q175 HD mouse models that were recapitulated in postmortem HD human caudate and putamen^14,63^, showing cell-type specific alterations in HD. In MSNs, mitochondrial dysfunction underlay a detrimental innate immune response^14^. Striatal OLs showed decreased expression of several markers, however, the correlation between mouse and human OL signatures was low in this case. Here, we show that OLs are increased in the human cingulate and caudate, and mouse and human OL show similar transcriptional dysregulation and reduced maturation. HD OLs are transcriptionally immature across multiple human and mouse brain regions. The fact that this phenotype spans the severely affected caudate, moderately involved cingulate, and the relatively preserved nucleus accumbens suggests that the deficits are independent of disease severity or anatomic region. Nonetheless, our data shows that impaired OL maturation is progressive with HD grade, and that in juvenile-onset HD, the maturation deficits largely involve OPCs. This was supported by ATACseq results demonstrating reduced binding of OL developmental TFs.

A caveat of our ATACseq analysis is that we FACS-sorted nuclei using NeuN. While this enriches for neurons in the sorted fraction, and glia in the flowthrough fraction, the analysis is limited by the sensitivity and specificity of the antibody we used. It is also known that not all neurons express NeuN^64^. Thus, our NeuN- fraction may still contain NeuN negative neurons. Furthermore, the NeuN- fraction may contain other non-glial cells such as endothelial cells. These caveats would be circumvented by the use of single cell ATACseq, a future direction for this study. That being said, our NeuN- fraction showed high read densities over the *Olig2* gene, which indicates that we indeed represented OL-lineage cells in the dataset.

The OL lineage pathology may reflect both a maturational deficiency and an inability to turn over myelin components. We suggest that OL defects start during the development of OLs from OPCs, which is consistent with previous studies^22^. In our human data, this finding was most pronounced in juvenile-onset HD, where maturational deficits appear to almost entirely involve OPCs, based on the pseudotime analysis. We speculate this may arise from the longer CAG repeat lengths in juvenile-onset HD, and the fact that HTT is expressed more highly in OPCs compared to OLs^65^. Other studies suggest that MYRF plays a role in OL maturational defects, Huang et al. showed that mHTT binds to MYRF and downregulates myelin genes^17^. MYRF is positively regulated by CHD7, which is regulated by OLIG2^49^—a master regulator of OL identity and a gene our results implicate in HD pathology. Finally, if the accumulation of mHTT downregulates the transcription of myelin genes, it may inhibit the ability of already myelinating OLs to produce myelin components during their normal turnover, thus leading to a progressive loss of myelin.

Metabolic disturbances in HD are hypothesized to lead directly to cellular distress, but less is known about their contributions towards epigenetic regulation, transcriptional deficits, and impact on cell maturation and identity. Both mouse and human snRNAseq data show dysregulation of key genes related to glucose and lipid metabolism that include genes that are within or downstream of several key metabolic pathways, including glycolysis, DAG, and hexosamine and protein glycosylation. Additionally, we show that DAG lipids, which activate PRKCE, were decreased in HD brains. Interestingly, protein kinase C signaling has been shown to be important to OPC differentiation, and myelination^66–69^. We found PRKCE levels to be decreased in HD, and that downregulating *PRKCE* in OPCs in vitro leads to increased differentiation of OLs. Further determination of the mechanism underlying these findings is the subject of future studies. Moreover, appropriate glucose metabolism is critical for the proper development and function of OLs, as OPCs transition to myelinating OLs^70–73^. Finally, thiamine metabolism is linked to OL differentiation based on evidence from deficient pyruvate dehydrogenase function in humans, which is known to cause structural white matter abnormalities^74^, and experimental evidence from pyruvate-dehydrogenase deficient mice, which show a reduction of O4-positive OL/OPCs^75^.

*TPK1* is a highly dysregulated gene and the most common DEG across cell types in the R6/2 12w striatal data. TPK1 regulates the conversion of thiamine to thiamine-pyrophosphate (TPP), a cofactor required for the conversion of pyruvate to acetyl-CoA, by alpha-ketoglutarate dehydrogenase in the TCA cycle and by ketolase in the pentose phosphate pathway, the latter being active in OL cultures and important for myelinating OLs^76^. Acetyl-CoA links metabolic processes to many epigenetic regulators of transcriptional control as it is used for histone acetylation, is required for the TCA cycle and for feeding metabolites into DNA and histone methylation, for generating both DAG and UDP-GlcNAc, and for PRKCE signaling and protein glycosylation by OGT (Fig. 8f). Interestingly, mutations in *TPK1* are linked to Thiamine Metabolism Dysfunction Syndrome 5, which pheno-copies HD, and mutations in thiamine transporters such as *SLC19A3* lead to biotin responsive basal ganglia disease^77^ which is treated with high T&B supplementation. Driven by our findings and similarities to other human disorders, we evaluated T&B treatment as a therapeutic strategy to reverse HD pathology in R6/1 mice. We hypothesized that TPK1 shows a compensatory increase in HD mice at later ages, responding to earlier metabolic changes, and tested this hypothesis by treating relatively pre-symptomatic R6/1 HD mice. Several transcriptional pathologies in HD were rescued by high dose T&B, suggesting promise as a potential treatment strategy. Excitingly, during the course of our study, a separate study was published showing a decrease in SLC19A3 and TPP in HD patients and in both R6/1 and zQ175 mice^78^. High dose T&B treatment produced both increased thiamine levels in the brain and CSF and behavioral rescue in R6/1 mice as early as 13 weeks. Our snRNAseq data revealed that R6/1 mice show maturation deficits and loss of cell identity genes similar to the R6/2 model and that treatment with T&B in the R6/1 mice, prior to *TPK1* or *SLC19A3* RNA changes, not only rescued a significant portion of dysregulated genes, including neuronal, but also specifically rescued expression of a specific subtype of inhibitory neurons and OPC and OL maturation genes. Furthermore, there was a reduction in the total number of significant DEGs in all cell types, except for in Ex neurons which may reflect compensatory changes due to the discordant levels in the genotype and treatment effects, but this requires further study outside the scope of this work. These data provide validation of the two studies and additional mechanistic insight that rescue by T&B likely acts in part through rescue of transcriptional deficits in many cell types and not just MSNs, whose survival was rescued in the other study, including a specific subpopulation of inhibitory neurons expressing *Adarb2*, and of OPC/OLs. Our data suggests that OL maturation impairments may be driven, in part, by thiamine metabolism and changes in the binding of TFs that regulate OL maturation, including SOX9 and 10 and OLIG1 and OLIG2. Furthermore, HD OPCs seem to have increased commitment into COP and immature OL which could be driven by decreased DAG and PRKCE, which is rescued by T&B treatment (Fig. 8g).

Lastly, these data further supports T&B as a viable potential treatment for HD, now undergoing a clinical trial in Spain (https://clinicaltrials.gov/ct2/show/NCT04478734), and supports the utility of using single cell approaches and CNM to guide therapeutic target identification and evaluation.

## Methods

All research conducted for this study complies with all relevant ethical regulations including IACUC approval protocol #s AUP-18-155 and, ASRC-2022-1 and the Columbia University IRB protocol # AAAT2895. Human brain tissues from HD and non-HD patient autopsies, which were diagnosed based on accepted neuropathological criteria, were obtained from the New York Brain Bank. All brains were donated after consent from the next of kin or an individual with legal authority to grant such permission. The use of postmortem brain tissues for research was approved by the Columbia University Institutional Review Board (IRB protocol # AAAT2895) with informed consent from patients or their families. The Institutional Review Board has determined that clinicopathologic studies on de-identified postmortem tissue samples are exempt from Human Subject Research according to Exemption 45 CFR 46.104(d)(2).

Mice: All experimental procedures were in accordance with the Guide for the Care and Use of Laboratory Animals of the NIH and animal protocols were approved by Institutional Animal Care and Use Committees at the University of California Irvine (UCI), an AAALAC accredited institution - PROTOCOL # AUP-18-155. Animal work for OPC cultures was approved by Institutional Animal Care and Use Committees at the Advanced Science Research Center at the City University of New York, an AAALAC accredited institution - PROTOCOL # ASRC-2022-1. R6/1 (Jax strain 006471 B6.Cg-Tg(HDexon1)61Gpb/J carrying CAG 115-150) and R6/2 (Jax strain 006494 B6CBA-Tg(Hdexon1)62Gpb/3J carrying CAG 120 +/− 5) mice have been described elsewhere in detail^31^. For the study using R6/2 mice, 10 five-week-old R6/2 and non-transgenic (NT) male mice were purchased from Jackson Laboratories and aged to 8 or 12 weeks. For the thiamine/biotin study using R6/1 mice, 10 five-week-old R6/1 and NT male and female mice were purchased from Jackson Laboratories. R6/1 mice (5/grp) were given a daily dose of combined 50 mg/kg thiamine and 20 mg/kg biotin (Caymen, Ann Arbor, MI) or vehicle (PBS) I.P. beginning at age 8 weeks, treated for 7 weeks, then euthanized at age 15 weeks. All mice were housed in groups of up to five animals/cage under a 12 h light/dark cycle with ad libitum access to chow and water at ambient temperature: 70F and 50% humidity. Mice were euthanized by pentobarbital overdose and perfused with 0.01 M PBS. Striatum and cerebral cortex were dissected out of each hemisphere and flash-frozen for snRNAseq or biochemical analysis.

### Single nuclei RNAseq

#### Mouse

Single nuclei were isolated from ½ hemisphere full striatal or full cortex in Nuclei EZ Lysis buffer (Cat#NUC101-1KT, Sigma-Aldrich) and incubated for 5 min. Samples were passed through a 70 μm filter and incubated in additional lysis buffer for 5 min and centrifuged at 500 × *g* for 5 min at 4 °C before two washes in Nuclei Wash and Resuspension buffer (1×PBS, 1% BSA, 0.2 U/μl RNase inhibitor). Nuclei were FACS sorted using DAPI to further isolate single nuclei and remove additional cellular debris. These nuclei were run on the 10× Chromium Single cell 3’ gene expression v3 platform. Libraries were QCed and sequenced on the NovaSeq 6000 using 30 bases for read 1 and 98 bases for read2, ß to obtain >=50 K reads per a cell. A total of 109,053 cells with 6.1 billion reads were sequenced for the 24 samples with on average 4544 cells per sample with ~55.6 K reads each. Alignment was done using the CellRanger pipeline v3.1.0 (10× Genomics https://github.com/10XGenomics/cellranger) to a custom pre-mRNA transcriptome built from refdata-cellranger-mm10-1.2.0 transcriptome using cellRanger mkref. UMI Count matrices were generated from BAM files using default parameters of cellRanger count command. The gene barcode matrices for each sample were imported into R using the Read10X function in the Seurat R package^79^ (v3.1.5). Replicates were combined using cellRanger aggr.

#### Human

Dissection of the cingulate cortex, caudate nucleus, and nucleus accumbens from frozen postmortem specimens was performed on material procured and preserved from autopsies on control as well as grade II and grade III HD. These samples were obtained from the New York Brain Bank. All cases had RNA integrity numbers of >7. Brain tissue measuring ~ 5 × 4 × 3 mm were dissected on a dry ice cooled stage and processed immediately as described below. A Table of the cases and controls used is provided in Supplementary Data 4. Nuclei were isolated as described in (ref. 88). Briefly, brain tissue was homogenized in a Dounce homogenizer with 12–15 strokes of the loose pestle and 12–15 strokes of the tight pestle on ice in a Triton X-100 based, sucrose-containing buffer. The suspension from each sample was filtered through a BD Falcon tube with a cell strainer cap (Becton Dickinson, cat. no. 352235), washed, re-filtered, washed, followed by a cleanup step using iodixanol gradient centrifugation as described in ref. 75. The nuclear pellet was then re-suspended in 1% BSA in nuclease-free PBS (containing RNAse inhibitors) and titrated to 600–1200 nuclei/μl. The nuclear suspensions were processed by the Chromium Controller (10× Genomics) using single Cell 3′ Reagent Kit v2 or v3 (Chromium Single Cell 3′ Library & Gel Bead Kit v2/v3, catalog number PN-1000075; Chromium Single Cell A Chip Kit, 48 runs, catalog number: 120236; 10× Genomics). *Sequencing and alignment:* Sequencing of the snRNAseq libraries was done on Illumina NOVAseq 6000 platformV4 150 bp paired end reads. Alignment was done using the CellRanger pipeline (10× Genomics) to GRCh38.p12 (refdata-cellrangerGRCh38–1.2.0 file provided by 10× genomics). Count matrices were generated from BAM files using default parameters of the DropEst pipeline^80^.

### QC and filtering

#### Mouse

Based on the distribution of number of genes detected in each cell and the distribution of number of UMIs, nuclei with less than 200 genes or more than 6000 genes were excluded from the downstream analyses. Nuclei with percent mitochondrial reads aligning to mitochondria genes of more than 2% were excluded. UMI counts were then normalized in Seurat 3.0 and top 2000 highly variable genes were identified using FindVariableFeatures function with variance stabilization transformation (VST).

#### Human

To remove low quality cells, we first used the combined quality calls from the CellRanger algorithm as well as the DropEst algorithm. This allowed us to retain more high-quality nuclei than either algorithm alone. Data QC was done using the scater package^81^. Nuclei with percent exonic reads from all reads in the range of less 75% were included. Nuclei with percent mitochondrial reads aligning to mitochondria genes of more than 14% were excluded. Genes were filtered by keeping features with >10 counts per row in at least in 31 cells. A temporary count slot was created by decontaminating the counts from ambient RNA by calling decontX() function with default parameters in R^82^. These counts were used for downstream clustering, but not differential gene expression analysis.

Combining multiple datasets from different sequencing batches and count normalization: Using the R package Seurat (version 4.06)^83^, the datasets were merged after controlling for sequencing batches (four batches). We integrated the lognormalized and scaled datasets in Harmony version 0.1. The Harmony reductions were then added to the merged Seurat object containing all datasets. The merged object was normalized using SCTransform function in Seurat accounting for batch and percentage mitochondrial reads^84^.

### Dimension reduction and clustering

#### Mouse

Based on the elbow plot, top 20 PCs were retained for seurat objects with all cell types and 15 for the OPC and OL analysis. These PCs were used in the downstream unsupervised clustering using a shared nearest neighbor Louvain modularity optimization to identify clusters of cells of the same type. Some of the identified clusters were comprised of multiple cell types, therefore we subclustered these cells for further downstream DEG generation and analysis (Supplementary Fig. 1a).

#### Human

Pre-clustering of nuclei was done in Seurat using the shared nearest neighbor smart local moving algorithm^85^ after using the iNMF or UMAP reducions, and calling FindClusters(…, algorithm=3,method = “igraph”, n.iter = 100, …). Several resolution and k options were trialed to select the option with the largest number of pre-clusters with the high lineage purity. Lineage identity was determined for each cluster using geneset enrichment analysis of lineage markers^86^ and by inspecting cluster markers generated by scran::findmarkers(direction = ”up”) function^87^. We also depended on the cell_classifier tool we previously used^88^. Pre-clusters with mixed identities based on enrichment of multiple lineage genes were sub-clustered iteratively until all pre-clusters showed pure identities which we combined into lineages (Astrocytes, neurons, oligodendrocytes, myeloid, endothelial, OPCs, and ependymal cells). Sub-clustering of select pre-clusters was done as needed to get the lineage-pure small clusters. We next combined the clusters of the same lineage to call the lineages presented in Fig. 1e.

After getting pure OL and OPCs, a new object from these cells only was created in monocle3. Corpus callosum cells were removed, because no HD corpus callosum samples were included in the dataset. Filtering lowly expressed genes yielded 16955 genes. The SCT normalized counts were used to reduce the dimensions using the PHATE function^89^ in R correcting for batch (using the mutual nearest neighbor option), and using the following parameters: KNN = 5, Dim=3, Decay=50, T = 10. Clustering was done in monocle3 utilizing the three PHATE reductions as input using the Levine algorithm.

### Cluster annotation and differential gene expression

#### Mouse

Unsupervised clustering was done using shared nearest neighbor Louvain modularity optimization. For each cluster, we used multiple cell type-specific marker genes that have been previously described in the literature to determine cell type/state identity. Exemplary genes used as markers for major cell types are shown in Supplementary Fig. 1. Differentially expressed genes between different clusters, ages or disease groups were identified using Wilcoxon Rank Sum test on genes that are expressed in at least 25% of the group. Further sub-clustering was conducted on some of the main clusters due to mixed cell types represented in that cluster, e.g., OPCs and premyelinating oligodendrocytes and astrocytes with vascular cells. Specifically, for subclustered OPCs and OL, OL-lineage, annotations were used from Marques et al.^37^ by looking at gene expression for marker genes identified in that study. These annotations were then collapsed into OPC and OL groups for ease of reference and consistency with human OPC and OL cells. Cluster and DEG analyses were conducted on each region and age for HD versus NT independently and combined where noted that the cells were integrated together across region and age.

#### Human

Differentially expressed genes (DEGs) between HD and control per anatomic region in OL and OPC separately were identified using EdgeR glmQLFTest adjusting for sequencing batch and using an FDR cutoff of 25% (9). The raw counts were used here, not the decontaminated counts. Retrieving the top 3000 differentially expressed genes resulted in adjusted p values less than 0.05, which were considered significant and were used for downstream analysis.

The CAG gene correlation analysis was conducted through the R package limma (version 3.14). Samples for the analysis were prepared using a pseudo-bulk approach. Gene expression data for each donor at a specific region were summed up together respectively to create pseudo-samples for the correlation analysis. Each pseudo donor-region sample were then log normalized and scaled using Seurat’s NormalizeData function (version 4.06) for optimal performance in limma. The covariates accounted for in the design matrix between samples included age and gender. Lastly, a row in the design matrix included the CAG repeats for each donor-region sample. The weights of the model were determined using limma’s lmFit with the arguments of the function including the pseudo-bulk donor region expression data and the design matrix as described above.

### Pseudotime trajectory analysis using Monocle3

#### Mouse

For oligodendrocyte developmental trajectory assessment, cells that were identified as OPC and OL lineage were used to create a separate Seurat object using SubsetData function on raw counts. Pseudotime analysis was conducted on the integrated data across all regions and ages.

#### Human

Pseudotime analysis was done using monocle3 employing the three PHATE dimensions to learn the principal graph using the following parameters: use_partition = F, learn_graph_control = list(euclidean_distance_ratio=0.5, geodesic_distance_ratio=0.7, minimal_branch_len=100, orthogonal_proj_tip=TRUE, rann.k = 100), close_loop = F). The root nodes were set as OPC cells. Grade 4 cases were excluded because after filtering low quality cells, two samples had very few OPCs after removing low quality cells and doublets.

### ATACseq

Isolation of NeuN+ and NeuN- nuclei: The pulverized tissue was resuspended in 2 ml NEB buffer (320 mM sucrose, 10 mM Tris-HCl pH 8, 5 mM CaCl_2_, 3 mM MgAc2, 0.1 mM EDTA, 0.1% Triton supplemented with protease inhibitors (Roche, 11836170001) and transferred through 40 μm tissue strainer, followed by 5 min centrifugation at 600 × *g* at 4 °C. The pellet was resuspended in 1 ml HS buffer (1.8 M sucrose, 10 mM Tris-HCl pH 8, 1 mM MgCl_2_ and Proteinase inhibitors) and centrifuged for 20 min at 16,000 × *g* at 4 °C. The nuclei containing pellet was resuspended in blocking buffer (PBS with 0.5% BSA, 5% Normal Goat Serum and Proteinase Inhibitors) and labeled with anti NeuN-PE antibody (1:100 dilution, Millipore, FCMAB317PE) and with Hoechst (1:2000 dilution, Invitrogen, H3570) for 30 min. The nuclei were filtered through 40 μm mesh and sorted using BD FACSAria™ with gates set to separate NeuN+ and NeuN- single nuclei populations. The nuclei were collected in tubes pre-coated with 1%BSA and sucrose was added to the sorted nuclei to a final concentration of 0.32 M followed by 15 min incubation on ice to stabilize the nuclei after sorting. The ATAC-seq was performed as described in Corces et al.^90^. Briefly, 50,000 sorted nuclei were transferred to tubes and pelleted by centrifugation at 2000 × *g* for 15 min. The pellet was resuspended in transposition reaction mix (25 μl 2× TD buffer, 2 μl transposase, 17 μl PBS, 0.5 μl 1% digitonin, 0.5 μl 10% Tween-20, 5 μl water) and incubated at 37 °C for 30 min following by clean up with Zymo DNA Clean and Concentrator kit (Zymo D4004). Illumina adapters were added by PCR to generate sequencing libraries as previously described. The ATAC-seq libraries were sequenced on an Illumina HiSeq 2000 for single-end 50 bp reads. Fastq files were aligned to the mm10 genome using Bowtie2 and paramaters previously described in Smith-Gearter et al. 2020^91^.

### Footprinting analysis

We used TOBIAS software^54^ for footprinting analysis of ATAC-seq data. Briefly, aligned BAM files were used to call accessible regions (peaks) using MACS2 using the following parameters:–nomodel–shift −100–extsize 200–broad. Peaks from all the samples across all conditions were merged to a set of union peaks using bedtools merge. TF motifs were downloaded from JASPAR CORE 2022 database (https://jaspar.genereg.net/). TOBIAS software robustly performs all steps of footprinting analysis including Tn5 bias correction, footprinting, and comparison between conditions and has been shown to outperform other common methods of footprinting. TOBIAS also calculates TF binding on a global level across all sites as well as the locus-specific level using JASPAR motif data. Scatterplots generated using ggplot2 (3.3.5).

### IRIS3

We used IRIS3^52^ to infer cell type specific regulons for single nucleus RNA-seq data from 12 week mouse striatal and cortical tissue. Specifically, gene count matrices from the full datasets were subset so that only OPC and OL cells are retained for striatal and cortical samples (4689, 3871 cells, respectively). These matrices together with sub cluster IDs were imported to the IRIS3 web portal (https://bmbl.bmi.osumc.edu/iris3/) and data were QC filtered, normalized and log transformed. DrImpute was used to impute the missing values. Gene modules were detected using QUBIC 2.0 and motif analysis was performed using MEME suite. CTSR(cell type specific regulons) were reported for each of the identified clusters within the OPC and OL population.

### BIRD

We predicted cell type specific chromatin accessibility from snRNA data using the BIRD algorithm (https://github.com/WeiqiangZhou/BIRD)^55^. Specifically, the human postmortem snRNA data (OPC and OL cells in caudate only) was first used for imputation of miss values in the 10× data with ALRA algorithm (https://github.com/KlugerLab/ALRA). To make predictions for each cell type (OPC and OL), we pooled cells within each cell type as input for BIRD. Predictions of chromatin accessibility were made using models for the human reference genome trained using 167 ENCODE RNA-seq samples. Prediction chromatin accessibility signals across both cell types in HD and CTR samples were converted to WIG format for visualization in genome viewers.

### Gene ontology, KEGG pathway, and TF enrichment analyses

#### Mouse

DEGs, gene modules members, and bnet gene members were used for further analyses using GOrilla for gene ontology enrichment analyses, KEGG pathway analysis, and LISA for TF enrichment analysis.

#### Human

Gene Ontology term enrichment analysis was done in gProfiler2 package in R^86^. The results of edgeR DEG was used as input and the following options: (ordered_query = T, significant = T, exclude_iea = T, underrep = F, evcodes = F, region_query = F, max_p_value = 1, min_set_size = 0, max_set_size = 100, min_isect_size = 5, correction_method = “gSCS”). Statistical significance was determined using the more conservative gSCS method 38 yielding adjusted p values. Terms with adjusted p values <0.05 were considered significant. The terms shown in the Figs. are selected based on ordering the results based on negative_log10_of_adjusted_p_value followed by the ratio of the shared of number of genes enriched in a term to that of the total number of genes in the GO term (desc(intersection_size/term_size)). Dotplots were generated using ggplot2 (3.3.5).

### Network modeling

#### Mouse

Weighted gene co-expression network analysis (WGCNA)^39^)was used to identify gene network modules from the mouse snRNAseq data. Normalized count data from Seurat 3.0 were first used for feature selection, filtering all genes without at least 1 count in 25% of all cells. Co-expression networks were then generated for NT data using WGCNA. Correlative module-trait relationships were used to identify gene network modules that were positively correlated with specific cell types used as input, and module preservation statistics were used to assess recapitulation of gene networks in R6/2 data. Bayesian network modeling: To identify causal relationships between cell type-specific gene subnetwork we used a bayesian network modeling approach using the R package BNLearn^92^. Probabilistic graphical modeling has been previously used to assess causal relationships between genes/proteins with great success in recapitulating known biological pathway interactions from single cell data^93^. Our approach took advantage of the co-expressed gene networks we previously identified to try and find causal relationships amongst these genes. To better interpret our data we chose to use input data from individual cell types, which were identified to be most correlated with each individual gene network module. The resulting causal network would be cell type-specific and easier to biological interpret. Features were chosen based on their inclusion within these gene modules and additionally genes were added based on differential expression between R6/2 and NT mice for each cell type-gene network module pair. For instance, we identified that the turquoise gene network module most highly correlated with our MSNs, these genes and DEGs found in both D1 and D2 MSNs were used as input from both 8 and 12w striatal and cortical data. HD and NT networks were separately generated to identify changes in network structure between disease and control. No priors were used as input for the structure learning. Using this input we constructed our Bayesian networks with a bootstrap approach using 50% of samples and 200 rounds. Due to the spasticity of single nuclei data, even after gene filtering, we chose to use an interval method for discretization, factoring input data into 3 breaks. For structure learning we utilized Bayesian Dirichlet likelihood-equivalence scoring and a hill-climbing algorithm for searching for network structures. An average network was generated from each output where the strength and direction (empirical frequency computed from the probability of each edges’ existence and direction) of each causal edge were greater than or equal to 0.85 and 0.5, respectively. HD and control networks were then merged to identify changes in network structure, of both nodes and edges.

### Primary oligodendrocyte culture

Mouse primary OPCs were isolated with immunopanning as described previously^94^. Briefly, cerebral cortices from C57BL/6 pups at P7 were digested in papain solution for 20 min at 37 °C, followed by titration and filtration. Cells were then sequentially incubated in three immunopanning dishes (2 negative selections with BSL1, followed by 1 positive selection with anti-mouse CD140a antibody (BD Bioscience, 558774). After positive selection, OPCs were trypsinized, plated onto PDL-coated culture dishes with SATO medium supplemented with growth factors (10 ng/mL PDGF-AA and 10 ng/mL bFGF), and maintained in a 37 °C, 5% CO_2_ incubator for further expansion.

### siRNA Transfection

Mouse primacy OPCs were seeded onto PDL-coated 6-well plate at a density of 2 × 10^5^ cells/well a day before transfection. Cells were transiently transfected with either siRNA targeting *Prkce* or non-targeting control (Origene, SR427452) at a final concentration of 30 nM using X-tremeGENE 360 Transfection Reagent (Roche, 8724105001). After 24 h of knockdown, cells were cultivated with either proliferating (supplemented with growth factors) or differentiation (supplemented T3, 60 ng/mL) media. After 3 days of proliferation and 5 days of differentiation, cells were harvested, and proteins were extracted and processed for western blot analysis.

### Qualitative lipidomic analysis of samples by electrospray triple Quadrupole mass spectrometry coupled with high performance liquid chromatography

Total lipids were extracted from frozen 40–70 mg human brain dissected as described above. Lipidomics profiling in mouse plasma and tissue samples was performed using Ultra Performance Liquid Chromatography-Tandem Mass Spectrometry (UPLC-MSMS). Lipid extracts were prepared from homogenized tissue samples using modified Bligh and Dyer method^95^, spiked with appropriate internal standards, and analyzed on a platform comprising Agilent 1260 Infinity HPLC integrated to Agilent 6490A QQQ mass spectrometer controlled by Masshunter v 7.0 (Agilent Technologies, Santa Clara, CA). Glycerophospholipids and sphingolipids were separated with normal-phase HPLC as described before^96^, with a few modifications. An Agilent Zorbax Rx-Sil column (2.1 × 100 mm, 1.8 µm) maintained at 25 °C was used under the following conditions: mobile phase A (chloroform: methanol: ammonium hydroxide, 89.9:10:0.1, v/v) and mobile phase B (chloroform: methanol: water: ammonium hydroxide, 55:39:5.9:0.1, v/v); 95% A for 2 min, decreased linearly to 30% A over 18 min and further decreased to 25% A over 3 min, before returning to 95% over 2 min and held for 6 min. Separation of sterols and glycerolipids was carried out on a reverse phase Agilent Zorbax Eclipse XDB-C18 column (4.6 × 100 mm, 3.5 μm) using an isocratic mobile phase, chloroform, methanol, 0.1 M ammonium acetate (25:25:1) at a flow rate of 300 μl/min. Quantification of lipid species was accomplished using multiple reaction monitoring (MRM) transitions^96,97^ under both positive and negative ionization modes in conjunction with referencing of appropriate internal standards: PA 14:0/14:0, PC 14:0/14:0, PE 14:0/14:0, PG 15:0/15:0, PI 17:0/20:4, PS 14:0/14:0, BMP 14:0/14:0, APG 14:0/14:0, LPC 17:0, LPE 14:0, LPI 13:0, Cer d18:1/17:0, SM d18:1/12:0, dhSM d18:0/12:0, GalCer d18:1/12:0, GluCer d18:1/12:0, LacCer d18:1/12:0, D7-cholesterol, CE 17:0, MG 17:0, 4ME 16:0 diether DG, D5-TG 16:0/18:0/16:0 (Avanti Polar Lipids, Alabaster, AL). Lipid levels for each sample were calculated by summing up the total number of moles of all lipid species measured by all three LC-MS methodologies, and then normalizing that total to mol%. The final data are presented as mean mol% with error bars showing mean ± S.E. Statistical comparisons were done using a one-way ANOVA and Tukey’s test for post hoc analysis. Only results on DAG are provided. Boxplots were generated using ggplot2 (3.3.5).

### Western blots

#### Mouse

Brain tissue was prepared for western blot analysis as follows: Soluble/Insoluble Fractionation: Striatal tissue was processed as described previously^98^. Total Fractionation: Isolated striatum or cortex was homogenized with 20 strokes of a potter-Elvenhjem glass tissue homogenizer in 1 mL modified RIPA buffer (50 mM Tris-HCl pH 7.4, 1% NP-40, 0.25% Na-deoxycholate, 150 mM NaCl, 1 mM EDTA) supplemented with one Pierce protease inhibitor mini tablet (Fisher Scientific A32953), 1 mM PMSF, phosphatase inhibitors 2 (Millipore Sigma, P5726) (1:1000) and 3 (Millipore Sigma P0044) (1:1000), 10 μg/mL aprotinin, and 10 μg/mL leupeptin. Lysates were sonicated then centrifuged at 16,000 rcf for 15 min, and 5–10 μg analyzed by western blot. Combined linear range was quantified on Empiria by analyzing a concentration gradient of protein (1.25, 2.5, 5, 10, and 20 μg per lane) with Revert for each antibody (Licor) to determine loading concentration. Protein was then subjected to SDS/PAGE on a NuPage Novex 4–12% Bis-Tris precast gel (Thermo Fisher NW04125) with MOPS running buffer (Invitrogen NP0001) and transferred onto a Immobilon-FL PVDF (Millipore Sigma IPFL00010) membrane. 5 µg of reduced, insoluble protein from Insoluble Fractions were resolved on 3–8% Tris-Acetate Poly-Acrylamide gels. Whole protein was quantified using the revert assay (LI-COR Biosciences 926-11016), and the membrane was blocked with Intercept (TBS) Blocking Buffer (LI-COR biosciences 927-60010) for 1 h. The membrane was then incubated in primary antibodies overnight, washed three times with TBS-0.1% Tween-20, and incubated for 1 h in Intercept block supplemented with 0.1% Tween-20 and near-infrared conjugated secondary antibodies. Membranes were imaged on a LI-COR scanner and quantified using Empiria Software. Experiments were performed at least twice with multiple biological replicates. Antibodies for the following antigens were used: DGKB (Thermofisher cat# PA5-15416 1:1000), PRKCE (Invitrogen PA5-83725 – 1:1000), p-PKCε (ser729) (Millipore 06-821-1; 1:1000), SGK1 (abcam - ab59337 1:1000), TPK1 (Fisherscientific cat# 50-172-6732 1:500), GPI1 (Thermofisher cat# PA5-26787 1:1000), Anti-Huntingtin Antibody (a.a. 1-82|MAB5492 - EMD Millipore 1:1000). The mice used for westerns were from two separate cohorts and did not include the mice used for snRNAseq and snATACseq. 6 males animals per group were used for each western except for the solb/insolb fractionated western which were mice were from a third cohort that included 4 male mice per group. All Western statistical analysis was performed using Students T-Test with two-tailed distribution and two-sample equal variance (homoscedastic). Exact *p*-values for significant differences are provided in the figure.

#### Human

Protein was extracted from dissected frozen tissue using RIPA buffer on ice. Protein concentration was estimated using a modified Bradford assay. Western blotting was performed using sodium dodecyl sulfate–polyacrylamide gel electrophoresis (SDS-PAGE) as described previously^99^. Briefly, protein lysates were separated by precast 4–20% Bis-Tris gradient gels (GenScript), followed by transferring onto PVDF membrane (Millipore). After 1 h blocking in blocking buffer (5% milk, 0.1% TBS-Tween) at room temperature, membranes were incubated overnight at 4 °C with primary antibodies. Antibodies for the following antigens were used: MAG (Proteintech cat#14386-1-AP - 1:1000-3000), MOG (Proteintech #12690-1-AP - 1:500-1000), PRKCE (Invitrogen PA5-83725 – 1:1000), p-PKCε (ser729) (Millipore 06-821-1; 1:1000), MBP (Cell signal #78896S, 1:1000), SGK1 (abcam - ab59337 1:1000), TPK1 (Fisherscientific cat# 50-172-6732 1:500), GAPDH (Proteintech 60004-1-Ig 1:1000), Actin (Proteintech 66009-1-Ig; 1:5000), Anti-mouse and anti-rabbit Peroxidase-AffiniPure Donkey IgG (H + L) (Jackson ImmunoResearch Labs Cat# 715-035-151 and 711-035-152). Detection was using enhanced chemiluminescence (cat# 1705061 or 1705062) on a Bio-Rad ChemiDoc™ Touch Imaging System. Band areas were normalized to Actin and/or GAPDH. Statistical comparisons were conducted using unpaired two-tailed t-test or Mann–Whitney test as appropriate. TPK1 was analyzed separately using similar methods as described in the mouse section and using only striatal tissue lysates. Boxplots were generated using ggplot2 (3.3.5).

Western blot analysis of OPC cultures was performed as outlined above with the following modifications. The following antibodies were used: rabbit anti-PRC-epsilon (Invitrogen PA5-83725, 1:1000), mouse anti-OLIG2 (Millipore, MABN50, 1:1000), mouse anti-CNPase (Biolegend, SMI-91, 1:5000), rabbit anti-MOG (Thermo, PA5-19602, 1:1000) and mouse anti-αTUBULIN (Calbiochem, CP06, 1:2500). Detection of target proteins was done by measuring chemiluminescence signal using ECL™ Prime Western Blotting Detection Reagent (Sigma, GERPN2232) on a ChemiDoc Imaging System (Bio-Rad). Image J was used to quantify the protein bands and αTUBULIN was used as loading control.

### Immunohistochemistry and in situ hybridization

Standard chromogenic and fluorescent immunohistochemistry as well as in situ hybridization were done as described previously^88^. Paraffin-embedded formalin-fixed tissue sections were used for IHC and ISH. The following antibodies were used CA2 (Abcam ab124687- 1:100), MBP (Invitrogen PA1-10008 – 1:5000). RNAscope™ was done per the manufacturer instructions using an RNAscope ™ multiplex Fluorescent v2 kit (ACDbio 323100) with the following probes for SPP1 (cat# 889751-C2), NEAT1 (cat# 411531-C3), and MBP (cat# 573051-C4).

### Imaging and quantification

Whole slides were scanned and the images on an Aperio™ Leica slide scanner at 40×. Fluorescent stained slides were scanned on Leica Aperio™ Versa scanner at 40×. additional images were taken on a Zeiss™ 810 LSM 800 confocal microscope at using a 40×/1.3 NA oil-immersion objective. For quantification of IHC, we employed an automated method using Qupath v0.2 positive cell detection algorithm^100^. Identification of pencil fibers and blood vessels was done using a pixel classifier trained on regions not quantified but in the same slide. Quantification of ISH slides uses positive cell detection method followed by subcellular detection. Only cells with nuclear signal were considered positive. Staining artifact and blood vessels were excluded. One or more images from each patient were used. The results were loaded in R v4.0. and cells with a minimum of 3 or more MBP dots or clusters were considered positive. NEAT1 and SPP1 were quantified in MBP positive cells. Nuclei with 2 or more dots or clusters were considered positive for SPP1 and with 2 or more dots/clusters for NEAT1. Statistical comparisons were done using one-tailed t-test or Wilcox rank test as appropriate. For calculating MBP:CA2 ratios, immunofluorescence for MBP and CA2 was performed on three or more images per case from 3 HD and 4 control caudate stained sections. The MBP signal was binarized using the threshold function in ImageJ (threshold detected automatically) and was divided by the number of CA2 positive cells counted in each image.

### Statistics and reproducibility

All features highlighted in the paper and reported as statistically significant have *p*-values <0.05 or adjusted *p*-values <0.1, unless otherwise stated. For single nucleus sequencing, we use negative binomial distribution to estimate power and sample size (https://satijalab.org/howmanycells). Assuming there are ≤18 cell types within the tissue, in order to detect rare cell types present at 1% with at least 20 nuclei per each type, we need at least 3775 nuclei to achieve power of 0.99. In our 10× experiment, we detected 4k–5k single nuclei per subject, to achieve power >0.99. No statistical methods were used to pre-determine sample sizes for the number of subjects needed but our sample sizes were similar to those reported in previous publications. No data were excluded from the analyses. The experiments were not randomized. For all human data collection and analysis, the investigators were blinded to conditions, data was unblinded after the completion of data collection. Mouse treatment groups were randomized but no other blinding was done.

### Reporting summary

Further information on research design is available in the Nature Portfolio Reporting Summary linked to this article.

## Supplementary information

Supplementary Information

Description of Additional Supplementary Files

Supplementary Dataset 1

Supplementary Dataset 2

Supplementary Dataset 3

Supplementary Dataset 4

Supplementary Dataset 5

Supplementary Dataset 6

Supplementary Dataset 7

Supplementary Dataset 8

Supplementary Dataset 9

Supplementary Dataset 10

Supplementary Dataset 11

Supplementary Dataset 12

Reporting Summary

## Data Availability

The human snRNAseq, mouse snRNAseq, and mouse ATACseq data generated in this study have been deposited in the GEO database under accession code GSE180928, GSE180294, and GSE180236, respectively. The aggregate imaging, western blot and DAG lipidomic data generated in this study are provided in the Source Data files. The JASPAR CORE 2022 data can be found at https://jaspar.genereg.net/. In addition to being found in the above locations, all raw data, materials, code, and associated protocols can also be requested from the corresponding authors and will be made available immediately to the requester. Source data are provided with this paper.

## Source data

Source Data

## Code availability

The code used in this study are deposited in the GitHub repository at https://github.com/jwuuci/snRNAseqHD.
