# Supplementary material for: Huntington disease oligodendrocyte maturation deficits revealed by single-nucleus RNAseq are rescued by thiamine-biotin supplementation
Source: Nat Commun. 2022 Dec 21;13:7791. doi: 10.1038/s41467-022-35388-x (PMC9772349; doi:10.1038/s41467-022-35388-x)
Supplement: Supplementary file 2 — Description of Additional Supplementary Files [file 41467_2022_35388_MOESM2_ESM.pdf]

Title: Supplementary Data 1.
Description: Mouse DEGs per cell type

Title: Supplementary Data 2.
Description: Integrated mouse OPC and OL DEGs

Title: Supplementary Data 3.
Description: Mouse WGCNA & cWGCNA gene members, WGCNA stats

Title: Supplementary Data 4.
Description: Causal network gene members and interactions, IRIS3, LISA.

Title: Supplementary Data 5.
Description: ATACseq Tobias differential binding results

Title: Supplementary Data 6.
Description: Human sample metadata and demographics

Title: Supplementary Data 7.
Description: Human cluster markers regression CAG by lineage

Title: Supplementary Data 8.
Description: Human DEGs OPC and OL by region, GO, and venn analysis

Title: Supplementary Data 9.
Description: Human Gene module scores by lineage grade levine clusters

Title: Supplementary Data 10.
Description: R61 genotype DEGs

Title: Supplementary Data 11.
Description: R61 TB v Veh DEGs

Title: Supplementary Data 12.
Description: R61 TB v NT DEGs
