# Supplementary material for: Huntington disease oligodendrocyte maturation deficits revealed by single-nucleus RNAseq are rescued by thiamine-biotin supplementation
Source: Nat Commun. 2022 Dec 21;13:7791. doi: 10.1038/s41467-022-35388-x (PMC9772349; doi:10.1038/s41467-022-35388-x)
Supplement: Supplementary file 15 — Reporting Summary [file 41467_2022_35388_MOESM15_ESM.pdf]

nature portfolio

Corresponding author(s): Leslie M. Thompson
James E. Goldman

Last updated by author(s): Nov 22, 2022

Reporting Summary

Nature Portfolio wishes to improve the reproducibility of the work that we publish. This form provides structure for consistency and transparency in reporting. For further information on Nature Portfolio policies, see our Editorial Policies and the Editorial Policy Checklist.

Statistics

For all statistical analyses, confirm that the following items are present in the figure legend, table legend, main text, or Methods section.

n/a	Confirmed	
☐	☒	The exact sample size (*n*) for each experimental group/condition, given as a discrete number and unit of measurement
☐	☒	A statement on whether measurements were taken from distinct samples or whether the same sample was measured repeatedly
☐	☒	The statistical test(s) used AND whether they are one- or two-sided<br>*Only common tests should be described solely by name; describe more complex techniques in the Methods section.*
☐	☒	A description of all covariates tested
☐	☒	A description of any assumptions or corrections, such as tests of normality and adjustment for multiple comparisons
☐	☒	A full description of the statistical parameters including central tendency (e.g. means) or other basic estimates (e.g. regression coefficient) AND variation (e.g. standard deviation) or associated estimates of uncertainty (e.g. confidence intervals)
☐	☒	For null hypothesis testing, the test statistic (e.g. *F*, *t*, *r*) with confidence intervals, effect sizes, degrees of freedom and *P* value noted<br>*Give P values as exact values whenever suitable.*
☐	☒	For Bayesian analysis, information on the choice of priors and Markov chain Monte Carlo settings
☒	☐	For hierarchical and complex designs, identification of the appropriate level for tests and full reporting of outcomes
☒	☐	Estimates of effect sizes (e.g. Cohen's *d*, Pearson's *r*), indicating how they were calculated

Our web collection on statistics for biologists contains articles on many of the points above.

Software and code

Policy information about availability of computer code

Data collection	Masshunter (7.0), Empiria (1.0), Bio-Rad ChemiDoc™ Touch Imaging System, Aperio™ Leica slide scanner, Qupath v0.2
Data analysis	Open source software including: CellRanger pipeline v3.1.0 (10X Genomics https://github.com/10XGenomics/cellranger), Seurat R package (v3.1.5, 4.06), BNlearn (4.6.1), Diffbind (3.2.3), EdgeR (3.34.0), monocle3 0.2.3, CCInx: (https://github.com/BaderLab/CCInx), WGCNA (1.69-81), DropEst (0.8.6-2), Scater (1.20.1), Liger (1.0), gProfiler2 (0.2.0), Limma (3.46.0), SVA (3.40.0), DGCA (1.0.2), igraph (1.2.6), ggplot2 (3.3.5). BIRD algorithm (https://github.com/WeiqiangZhou/BIRD, ALRA algorithm (https://github.com/KlugerLab/ALRA), IRIS3 web portal (https://bmbl.bmi.osumc.edu/iris3/), TOBIAS (0.12.3, REF: https://doi.org/10.1038/s41467-020-18035-1), bnlearn (4.8.1), Harmony (1.0).<br><br>The code used in this study are deposited in the GitHub repository at https:github.com/jwuuci/snRNAseqHD.

For manuscripts utilizing custom algorithms or software that are central to the research but not yet described in published literature, software must be made available to editors and reviewers. We strongly encourage code deposition in a community repository (e.g. GitHub). See the Nature Portfolio guidelines for submitting code & software for further information.

Data

Policy information about availability of data

All manuscripts must include a data availability statement. This statement should provide the following information, where applicable:

- Accession codes, unique identifiers, or web links for publicly available datasets
- A description of any restrictions on data availability
- For clinical datasets or third party data, please ensure that the statement adheres to our policy

The human snRNAseq, mouse snRNAseq, and mouse ATACseq data generated in this study have been deposited in the GEO database under accession code GSE180928, GSE180294, and GSE180236, respectively (https://www.ncbi.nlm.nih.gov/geo/query/acc.cgi?acc=GSE180928, https://www.ncbi.nlm.nih.gov/geo/query/acc.cgi?acc=GSE180294, https://www.ncbi.nlm.nih.gov/geo/query/acc.cgi?acc=GSE180236). The aggregate imaging, western blot and DAG lipidomic data generated in this study are provided in the Source Data files. The JASPAR CORE 2022 data can be found at https://jaspar.genereg.net/. In addition to being found in the above locations, all raw data, materials, code, and associated protocols can also be requested from the corresponding authors and will be made available immediately to the requester.

Human research participants

Policy information about studies involving human research participants and Sex and Gender in Research.

Reporting on sex and gender	Information on sex was collected from the brain donors. Both males and females were included and data is available for each donor individually.
Population characteristics	Post-mortem human brains were obtained from the New York Brain Bank. the demographic information of the donors is provided in supplementary table-1
Recruitment	The donors donated the brains for scientific research. These include patients who were followed up by Neurologists at CIUMC and other institutions who referred the patients for brain donation. The donors knew the diagnosis before death, and there is no selection bias.
Ethics oversight	Human brain tissues from HD and non-HD patient autopsies, which were diagnosed based on accepted neuropathological criteria, were obtained from the New York Brain Bank. All brains were donated after consent from the next of kin or an individual with legal authority to grant such permission. The use of postmortem brain tissues for research was approved by the Columbia University Institutional Review Board (IRB protocol # AAAT2895) with informed consent from patients or their families. The Institutional Review Board has determined that clinicopathologic studies on de-identified postmortem tissue samples are exempt from Human Subject Research according to Exemption 45 CFR 46.104(d)(2).

Note that full information on the approval of the study protocol must also be provided in the manuscript.

Field-specific reporting

Please select the one below that is the best fit for your research. If you are not sure, read the appropriate sections before making your selection.

☒ Life sciences ☐ Behavioural & social sciences ☐ Ecological, evolutionary & environmental sciences

For a reference copy of the document with all sections, see nature.com/documents/nr-reporting-summary-flat.pdf

Life sciences study design

All studies must disclose on these points even when the disclosure is negative.

Sample size	For single nucleus sequencing, we use negative binomial distribution to estimate power and sample size(https://satijalab.org/howmanycells). Assuming there are <=18 cell types within the tissue, in order to detect rare cell types present at 1% with at least 20 nuclei per each type, we need at least 3775 nuclei to achieve power of 0.99. In our 10x experiment, we detected 4k-5k single nuclei per subject, to achieve power > 0.99.<br><br>No statistical methods were used to pre-determine sample sizes for the number of subjects needed but our sample sizes were similar to those reported in previous publications.
Data exclusions	No data was excluded
Replication	For all experiment, statistical analyses used a minimum of three true biological replicates. Where appropriate experiments were repeated 2 or more times and stated within the manuscript, e.g. for mouse western analyses each western and antigen quantification were repeated twice on separate cohorts of mice from the snRNAseq data. 6 mice were used for the initial western study and 4 mice for the fractionated westerns. All replication was successful and statistically significant. Mouse OPC culture experiments were replicated successfully by repetition (total n = 3). For human snRNAseq studies, replication was done by running the analysis on different brain regions and finding the same effect on

	oligodendrocyte differentiation.
Randomization	Randomization was used for mouse treatment groups for thiamine and biotin dosing
Blinding	For human data collection and analysis the investigators were blinded to conditions. No blinding was done for mouse data.

Reporting for specific materials, systems and methods

We require information from authors about some types of materials, experimental systems and methods used in many studies. Here, indicate whether each material, system or method listed is relevant to your study. If you are not sure if a list item applies to your research, read the appropriate section before selecting a response.

Materials & experimental systems

n/a	Involved in the study
☐	☒ Antibodies
☒	☐ Eukaryotic cell lines
☒	☐ Palaeontology and archaeology
☐	☒ Animals and other organisms
☒	☐ Clinical data
☒	☐ Dual use research of concern

Methods

n/a	Involved in the study
☒	☐ ChIP-seq
☒	☐ Flow cytometry
☒	☐ MRI-based neuroimaging

Antibodies

Antibodies used	Antibodies for the following antigens were used DGKB (ThermoFisher #PA5-15416 1:1000), PRKCE (Invitrogen #PA5-83725 – 1:1000), p-PKCε (ser729) (Millipore #06-821-1; 1:1000), SGK1 (abcam - #ab59337 1:1000), TPK1 (Fisherscientific # 50-172-6732 1:500), GPI1 (ThermoFisher #PA5-26787 1:1000), Anti-Huntingtin Antibody, (a.a. 1-82 | #MAB5492 - EMD Millipore; 1:1000). MAG (Proteintech #14386-1-AP - 1:1000-3000), MOG (Proteintech #12690-1-AP - 1:500-1000), , MBP (Cell signal #78896S, 1:1000), SGK1 (abcam - #ab59337 1:1000), GAPDH (Proteintech #60004-1-Ig 1:1000), Actin (Proteintech #66009-1-Ig; 1:5000), OLIG2 (Millipore, #MABN50, 1:1000), CNPase (Biolegend, #SMI-91, 1:5000), MOG (ThermoFisher, #PA5-19602, 1:1000), αTUBULIN (Calbiochem, CP06, 1:2500), Anti-mouse and anti-rabbit Peroxidase-AffiniPure Donkey IgG (H+L) (Jackson ImmunoResearch Labs # 715-035-151 and 711-035-152).
Validation	All primary antibodies were commercially purchased, and the validation information for each antibody from the manufacturers' websites includes: DGKB validated by Western blot (WB), Immunohistochemistry (IHC); PRKCE, WB, IHC; p-PKCε, WB; SGK1, WB, IHC, Immunocytochemistry-fluorescence (ICC/IF); TPK1, WB, IHC; GPI1, WB, IHC; Anti-Huntingtin Antibody, WB; MAG, WB, IHC; MOG, WB, IHC; MBP, WB, IHC, ICC/IF; GAPDH, WB, ICC/IF; Actin, WB, IHC, ICC/IF; OLIG2, WB, ICC/IF; CNPase, WB, IHC; αTUBULIN, WB, ICC/IF; Anti-mouse and anti-rabbit Peroxidase-AffiniPure Donkey IgG Immunoelectrophoresis and/or ELISA.

Animals and other research organisms

Policy information about studies involving animals; ARRIVE guidelines recommended for reporting animal research, and Sex and Gender in Research

Laboratory animals	Male mice strain B6CBA-Tg(HDexon1)62Gpb/3J Stock No: 006494 | B6CBA-R6/2 (CAG 120 +/- 5) were used at ages 8 and 12 weeks for the study. R6/1 mice (006471 B6.Cg-Tg(HDexon1)61Gpb/J carrying CAG 115-150), 10 five-week-old R6/1 and NT male and female mice (5/group) were purchased from Jackson Laboratories. Animals were housed at ambient temperature: 70F and 50% humidity.<br><br>For C57BL/6 mice 3 male and 3 female pups at postnatal day 7 were combined and used for every biological replicate.
Wild animals	No wild animals were used in the study
Reporting on sex	Both males (3) and females (3) were included for isolation of primary OPCs and combined per replicate experiment (n=3). For R6/2 snRNAseq and ATACseq only male mice were used in the study. For R6/1 both male and female mice were used. Gender was accounted for during statistical analysis where appropriate.
Field-collected samples	None collected
Ethics oversight	All experimental procedures were in accordance with the Guide for the Care and Use of Laboratory Animals of the NIH and animal protocols were approved by Institutional Animal Care and Use Committees at the University of California Irvine (UCI), an AAALAC accredited institution - PROTOCOL # AUP-18-155. Animal work for OPC cultures was approved by Institutional Animal Care and Use Committees at the Advanced Science Research Center at the City University of New York, an AAALAC accredited institution - PROTOCOL # ASRC-2022-1.

Note that full information on the approval of the study protocol must also be provided in the manuscript.
