# Supplementary material for: Huntington disease oligodendrocyte maturation deficits revealed by single-nucleus RNAseq are rescued by thiamine-biotin supplementation
Source: Nat Commun. 2022 Dec 21;13:7791. doi: 10.1038/s41467-022-35388-x (PMC9772349; doi:10.1038/s41467-022-35388-x)

Figure 7B - PKCe – GAPDH, p-PKCe – GAPDH (Caudate)

0215-3 p-PKCε - GAPDH

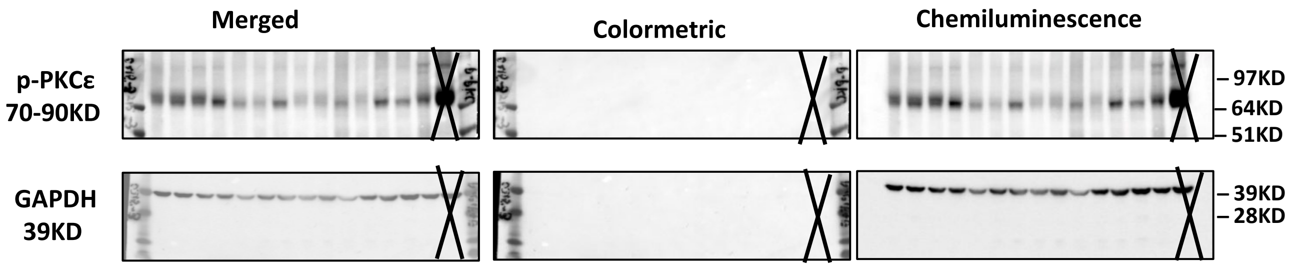

0105-3 PKCε / GAPDH

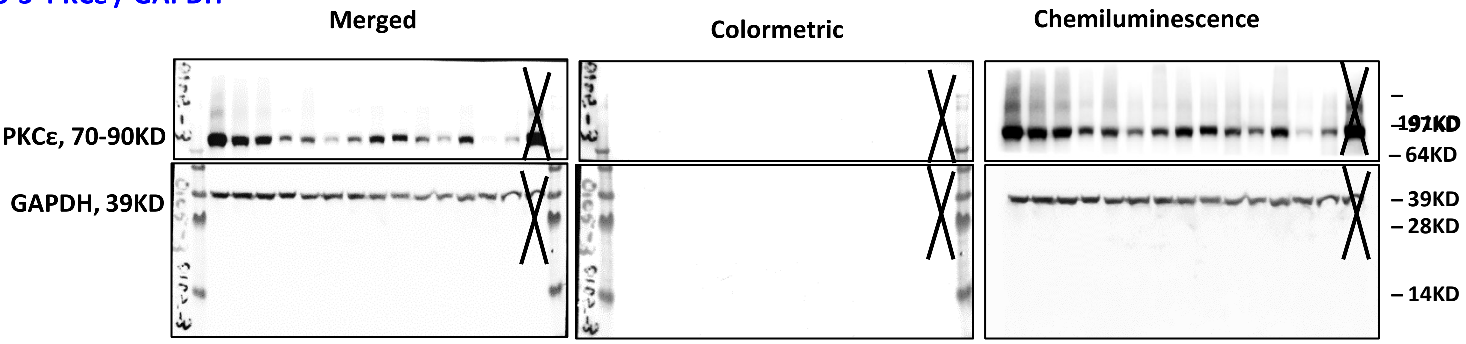

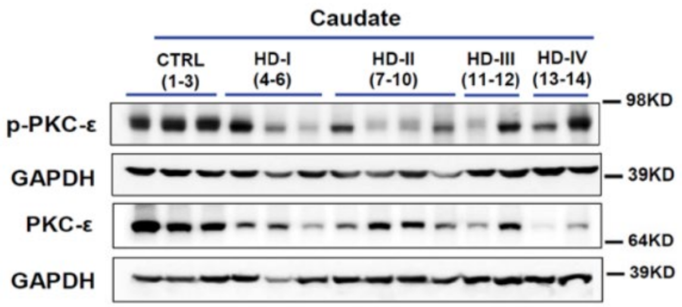

“X” indicates samples that are of a different condition not related to the study

Figure 7B - PKCe – GAPDH, p-PKCe – GAPDH (Cingulate)

0210-2 p-PKCε / GAPDH

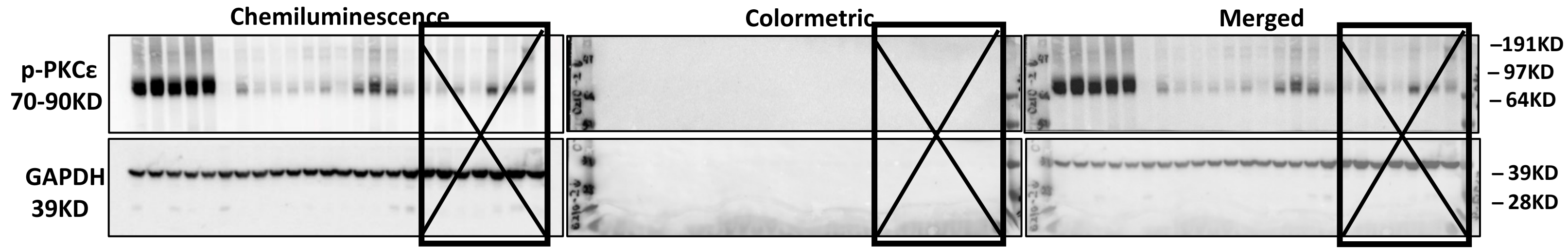

0210-3 PKCε / GAPDH (membrane blotted with AKT (~50KD, mouse) first, then stripped, blotted for PKCε (>60KD, rabbit)

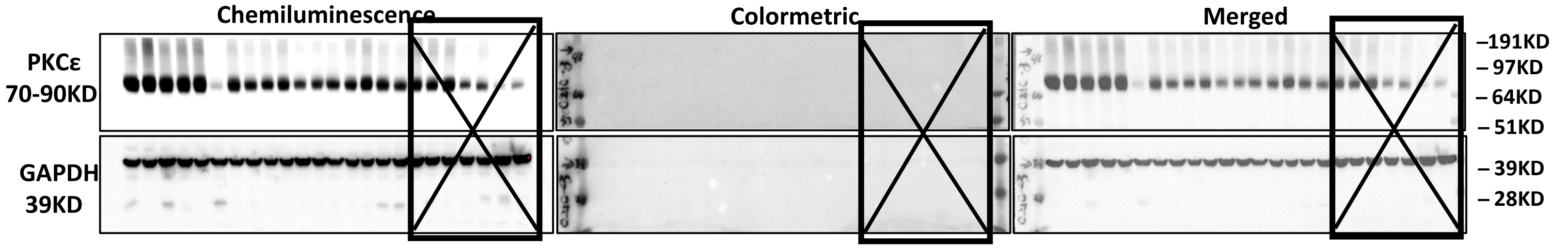

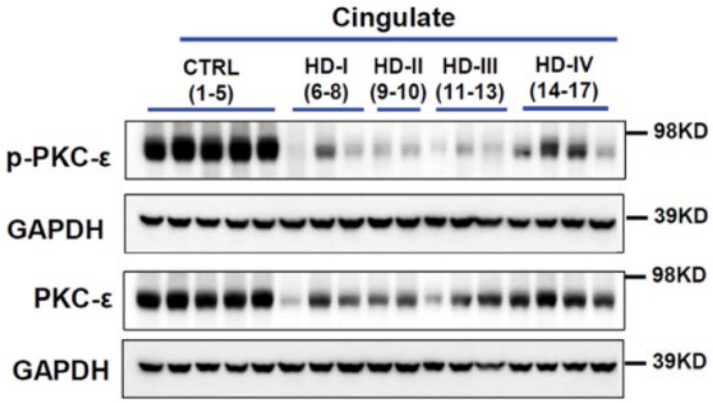

"X" indicates samples that are of a different condition not related to the study

Figure 7d - Prkce – Revert, p-Prkce – Revert (cortex)

Prkce pPrkce Cortex R6/2

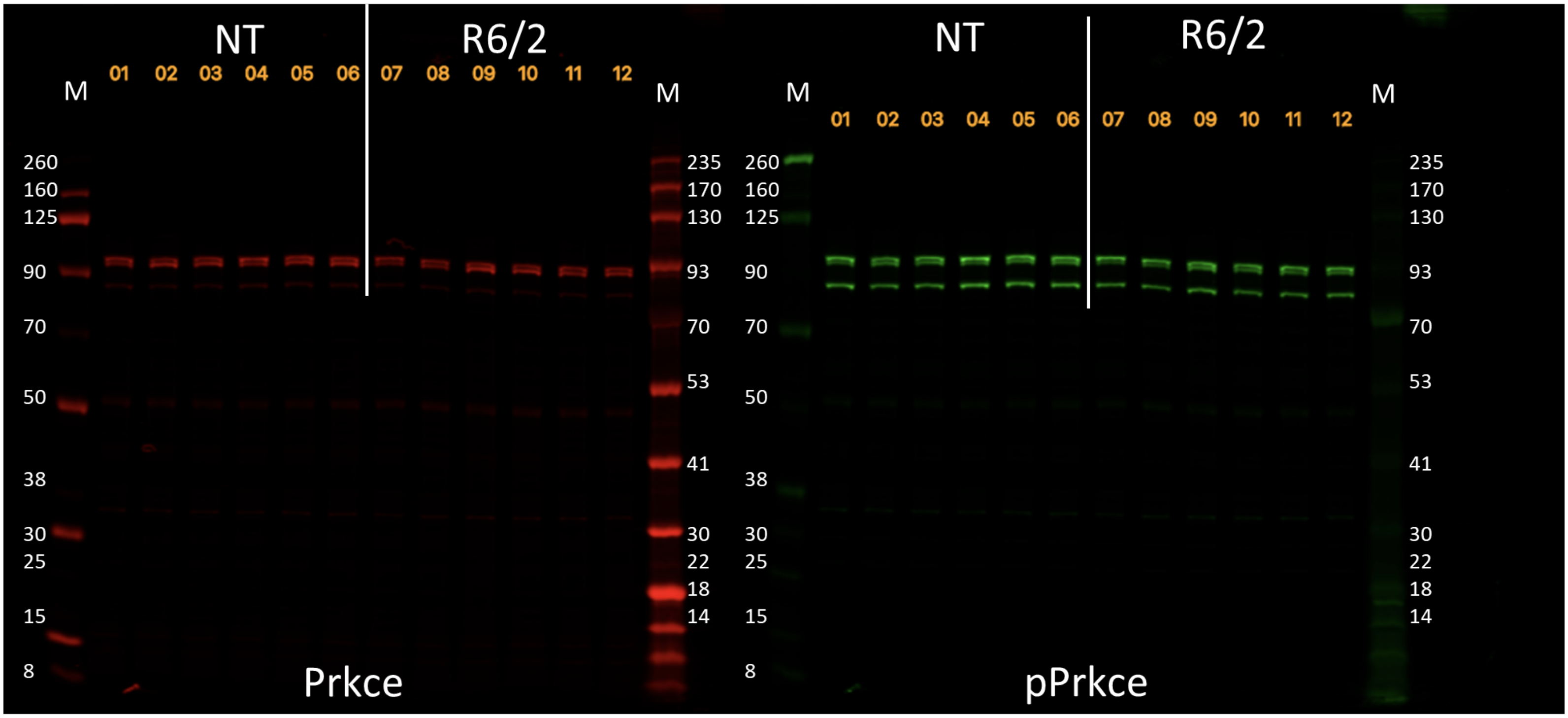

Prkce pPrkce Cortex R6/2

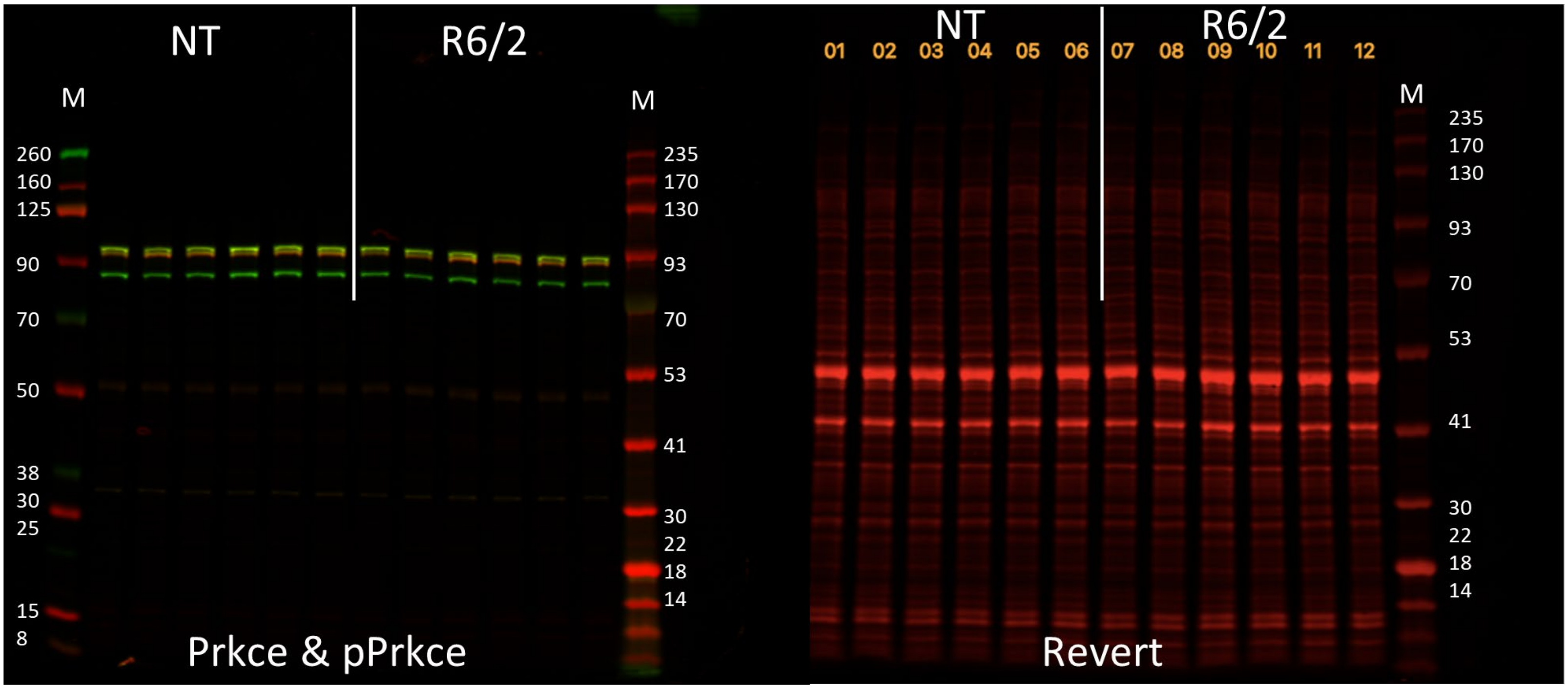

Figure 7d - Prkce – Revert, p-Prkce – Revert (striatum)

Prkce pPrkce Striatum R6/2

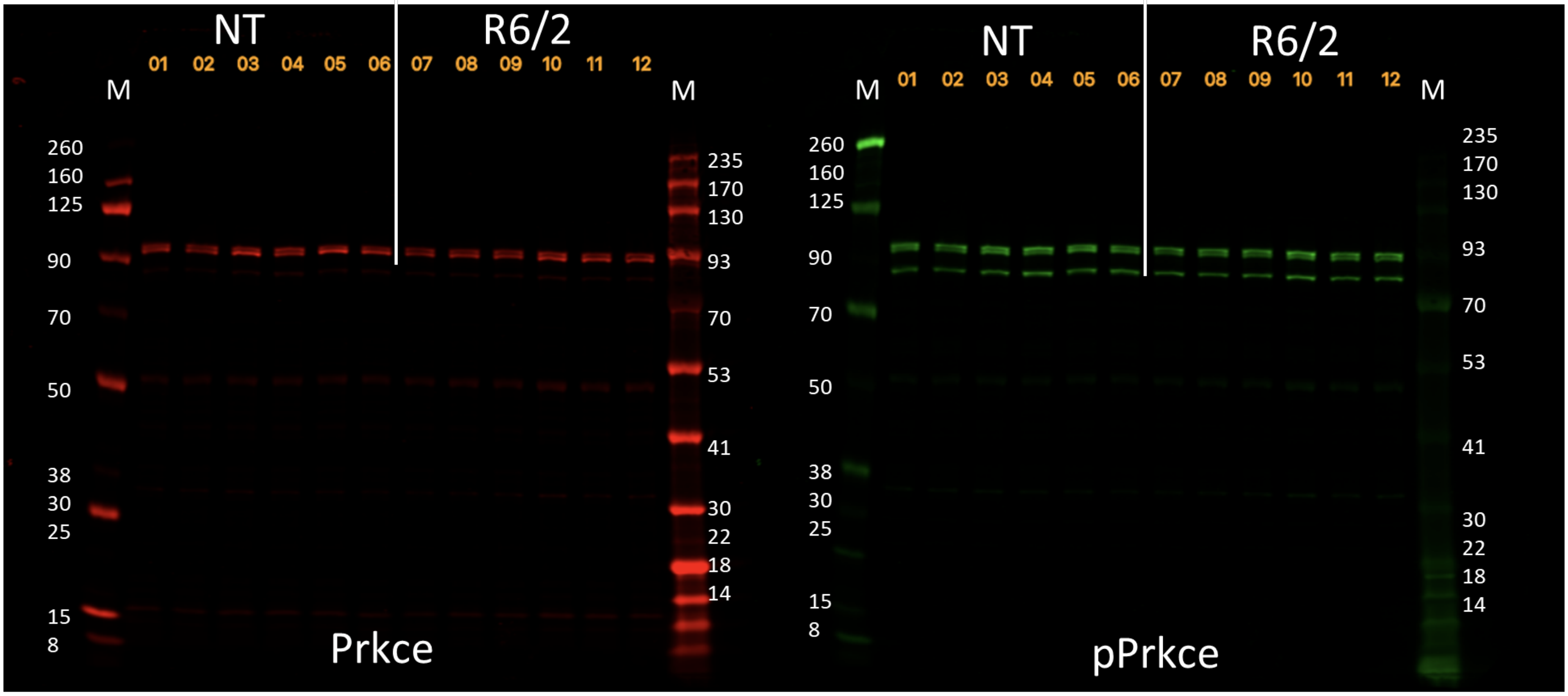

Prkce pPrkce Striatum R6/2

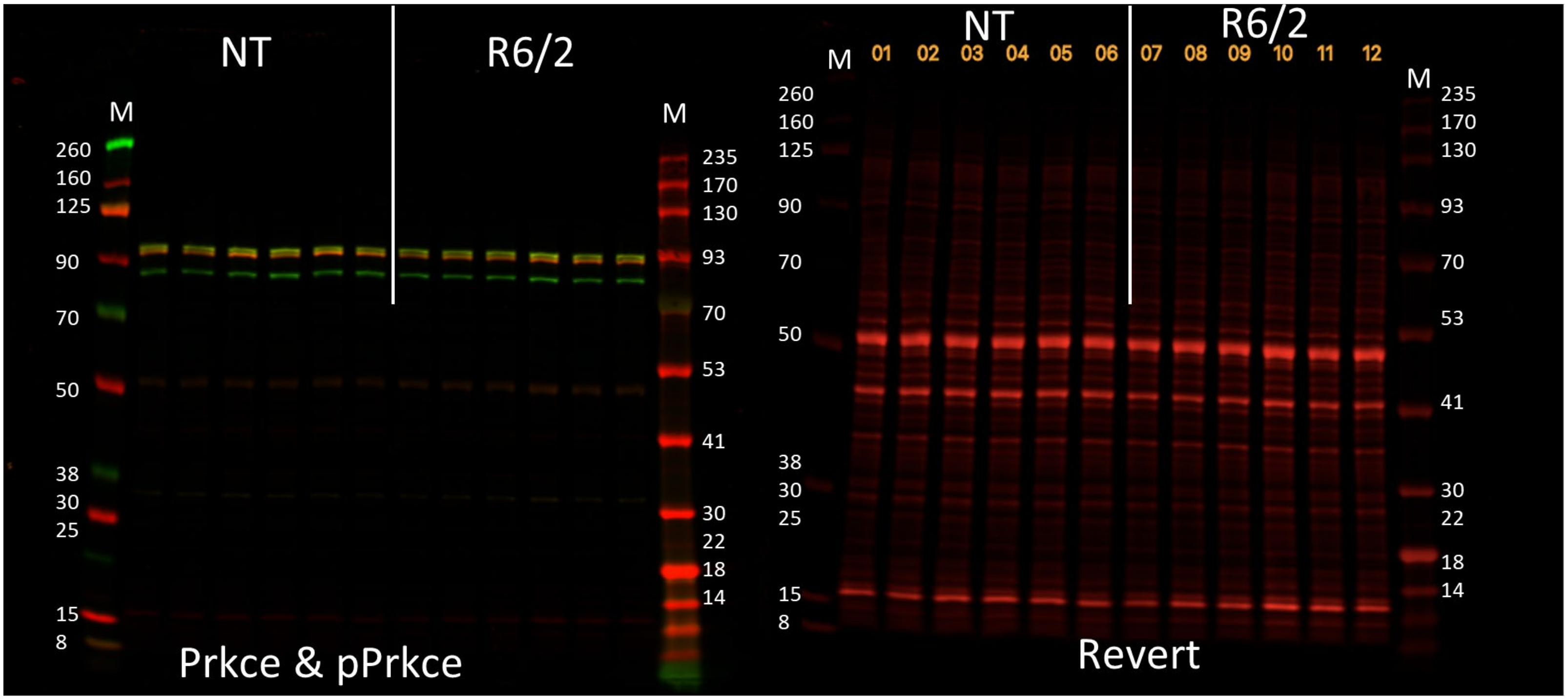

Figure 7d – Tpk1 – Revert, (cortex & striatum)

TPK1 Cortex R6/2

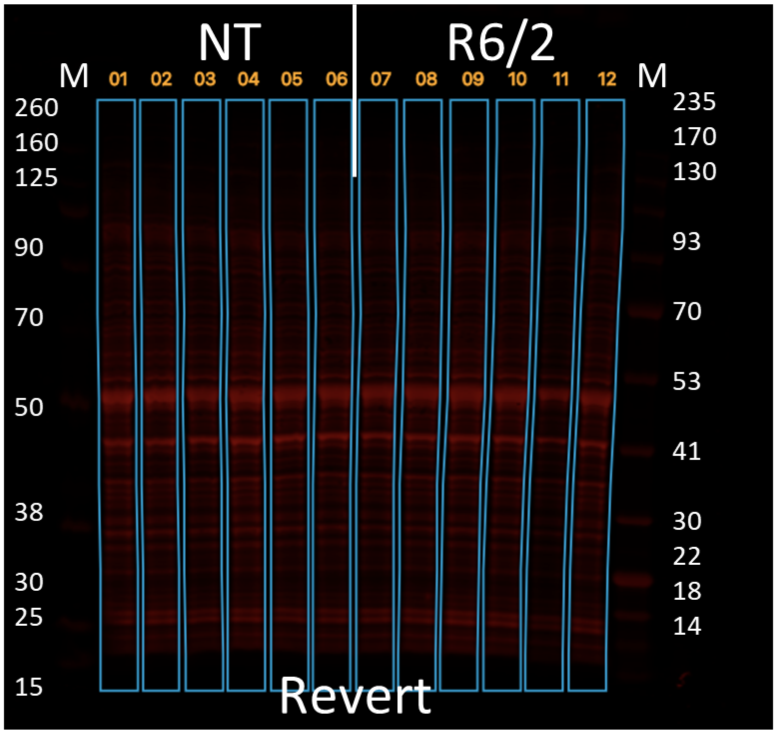

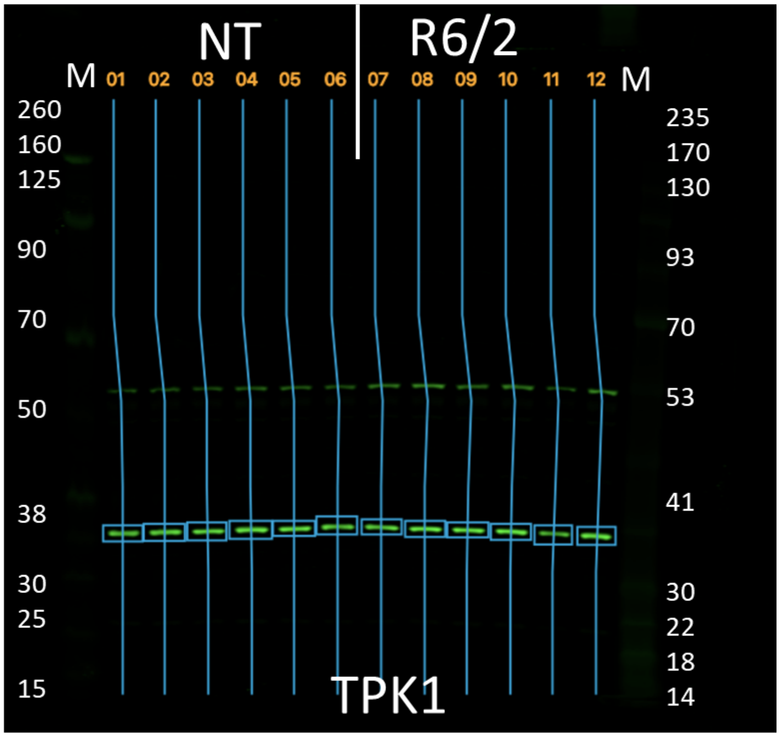

TPK1 Striatum R6/2

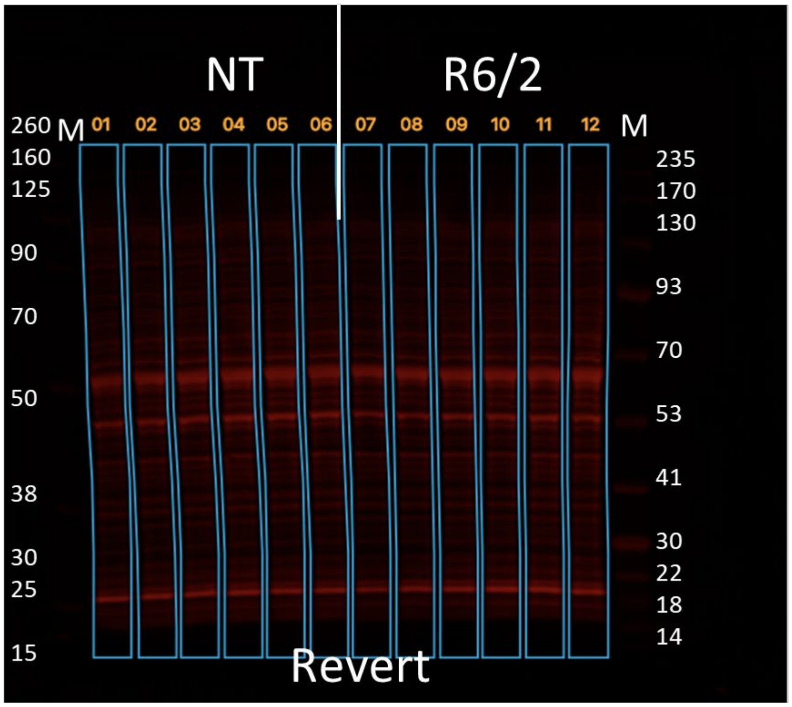

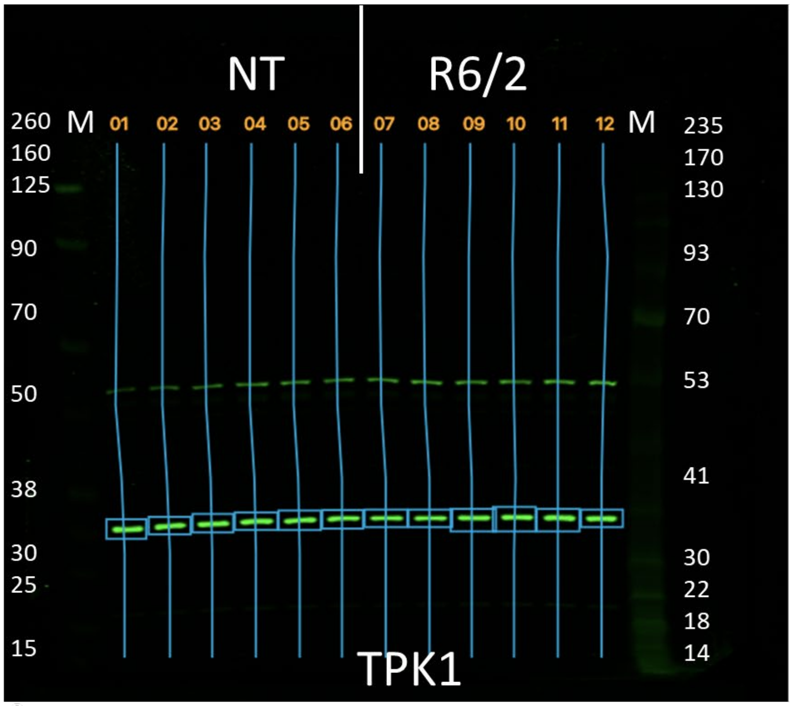

Figure 7f – Tpk1 – Revert, (human)

TPK1 human

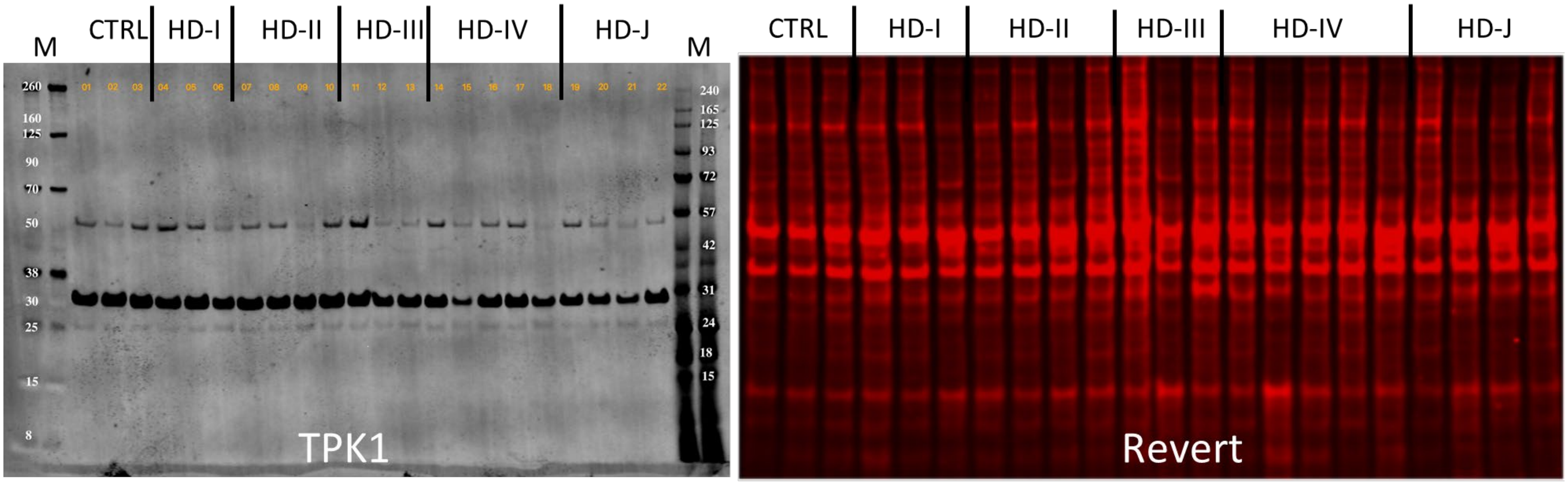

Figure 7i – in vitro WB –A-TUBULIN

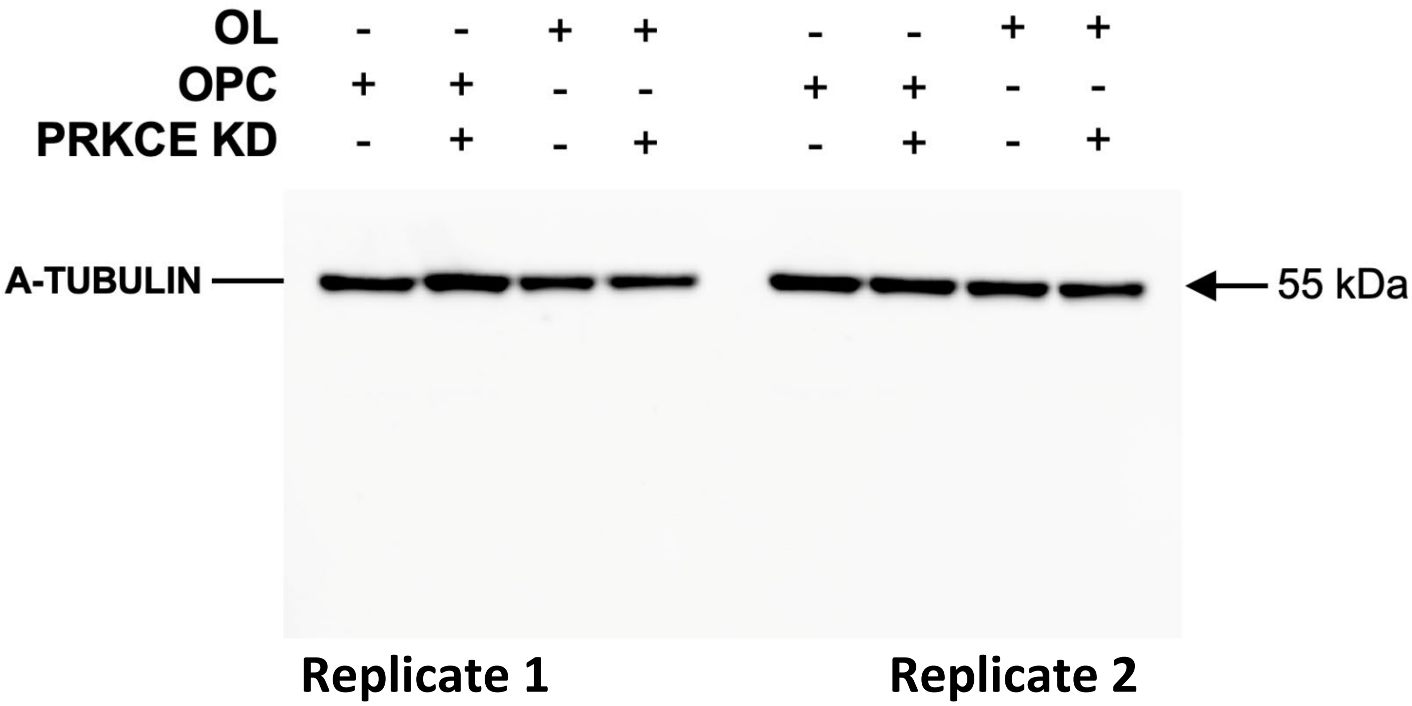

Figure 7i – in vitro WB CNPase

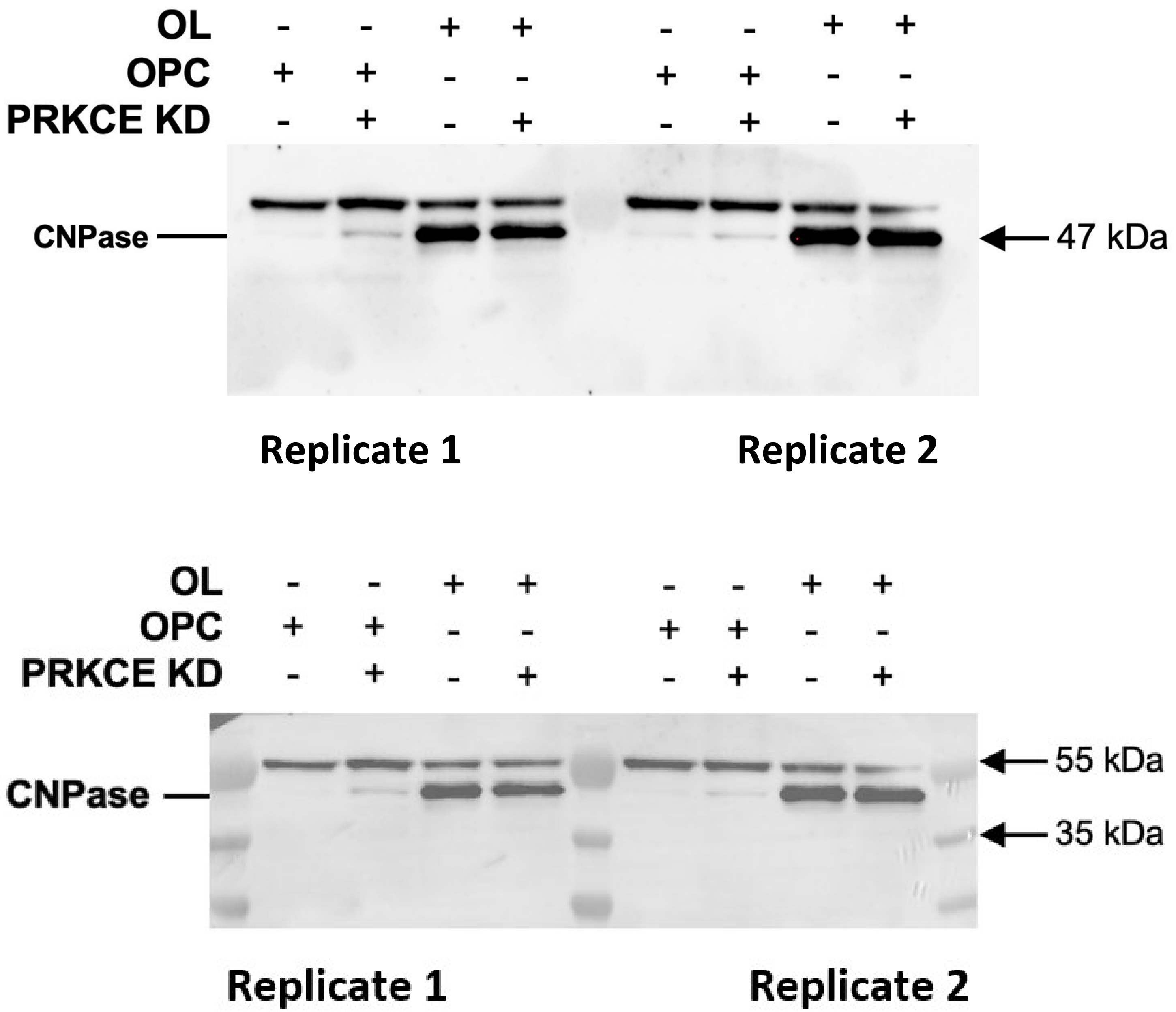

Figure 7i – in vitro WB MOG

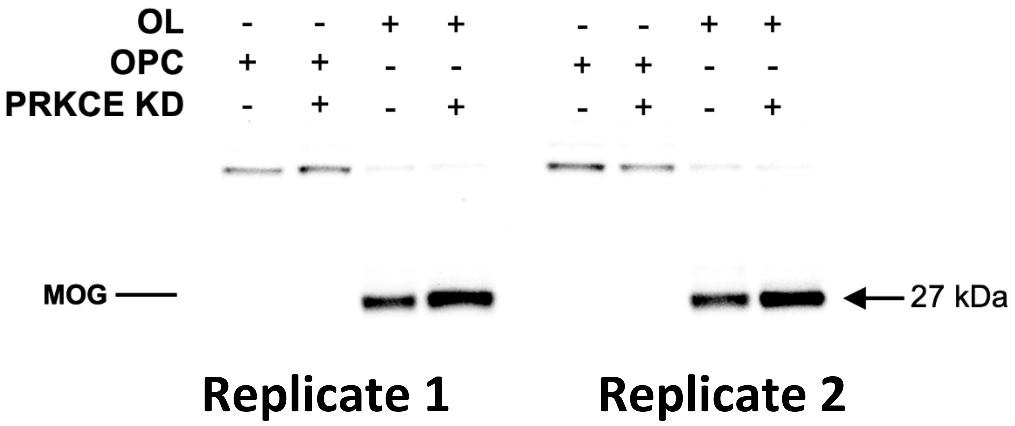

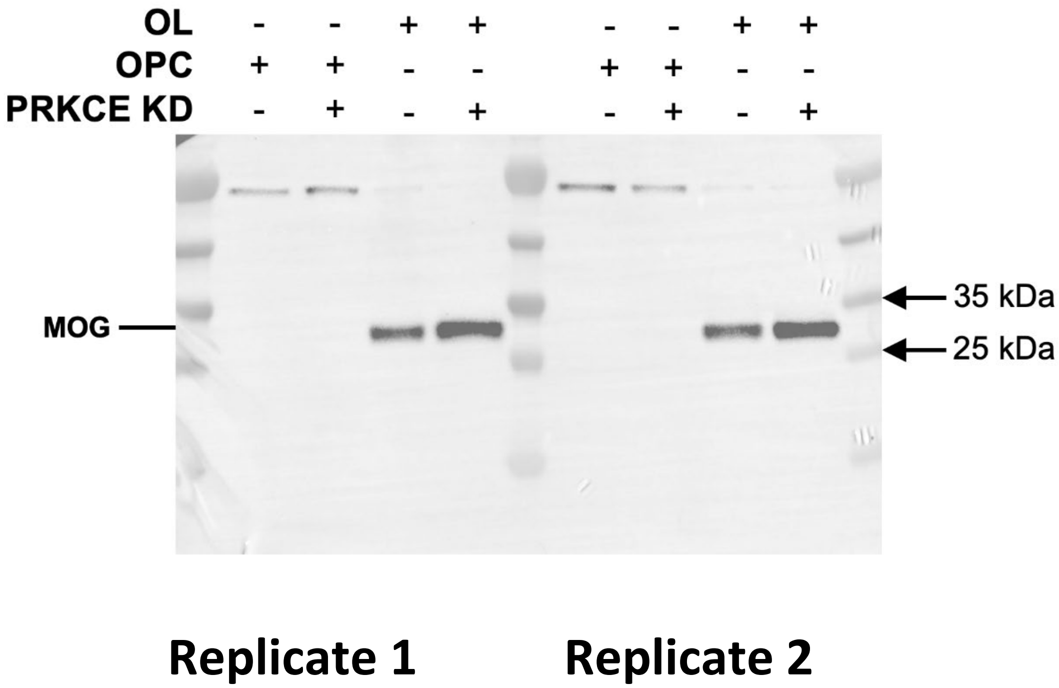

Figure 7i – in vitro WB OLIG2

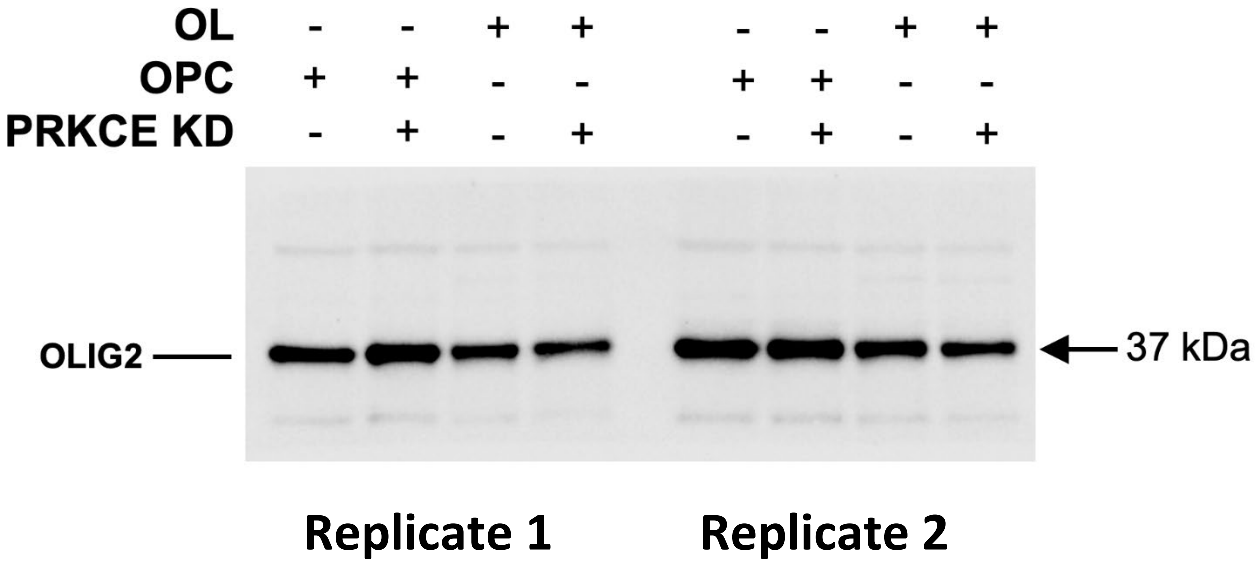

Figure 7i – in vitro WB PRKCE

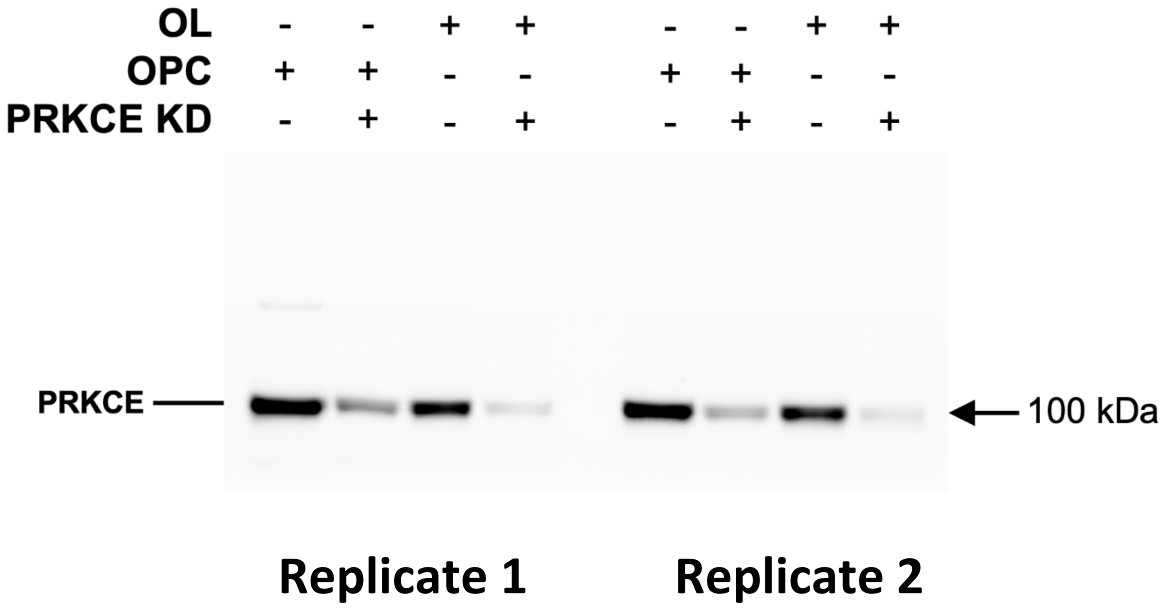

Figure 7i – in vitro WB A-TUBULIN

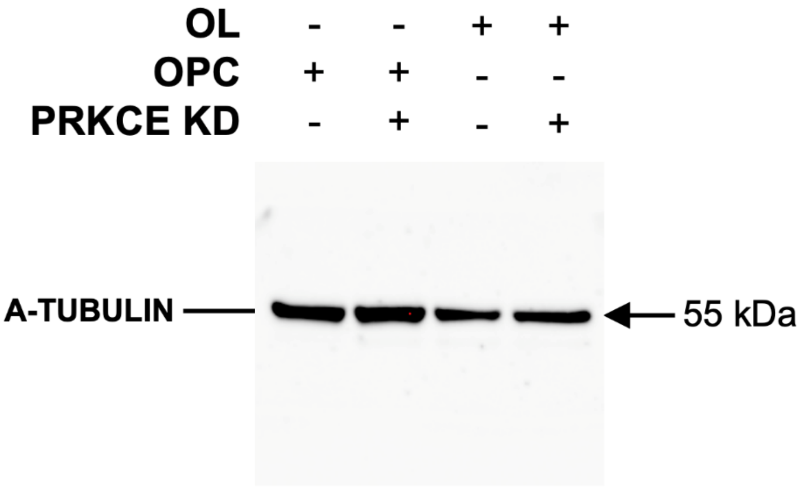

Replicate 3

Figure 7i – in vitro WB B-ACTIN

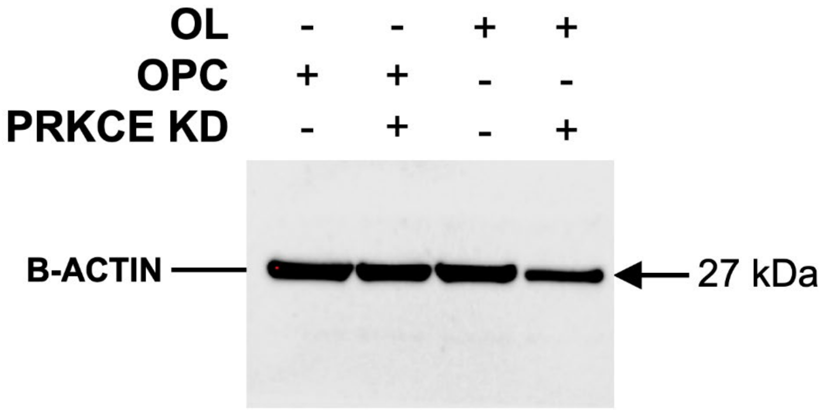

Replicate 3

Figure 7i – in vitro WB PRKCE

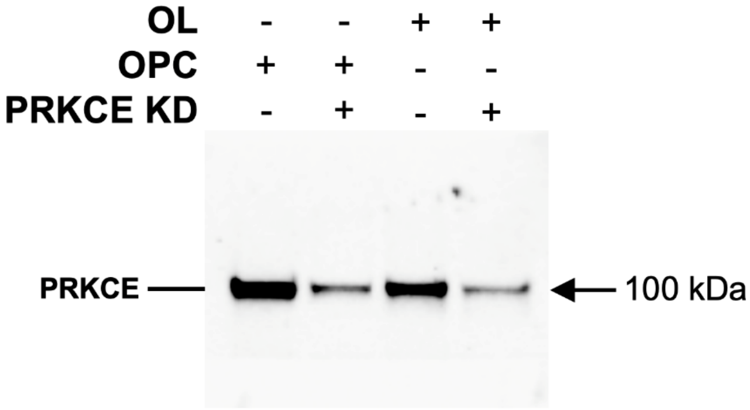

Replicate 3

Figure 7i – in vitro WB MOG

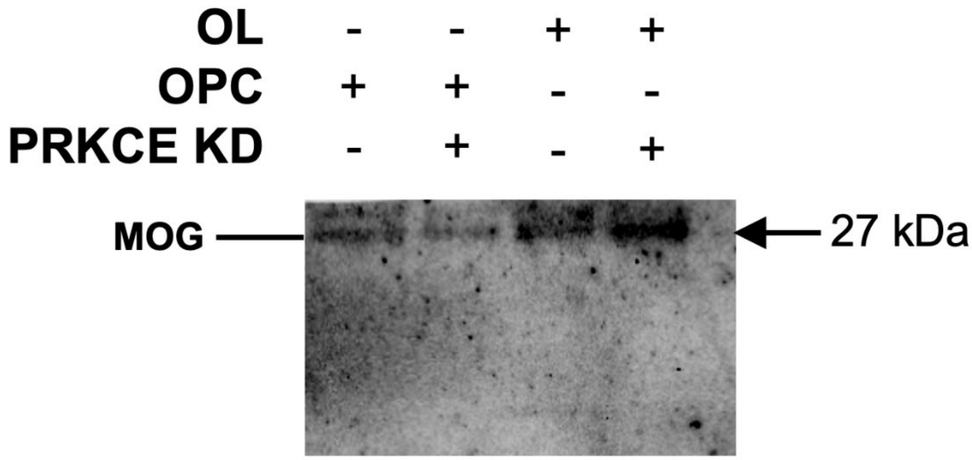

Replicate 3

Original

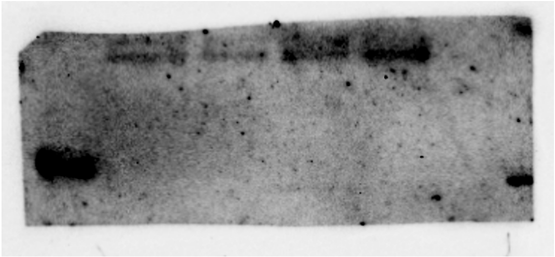

Figure S8B - 0215-1 MAG – GAPDH - MBP

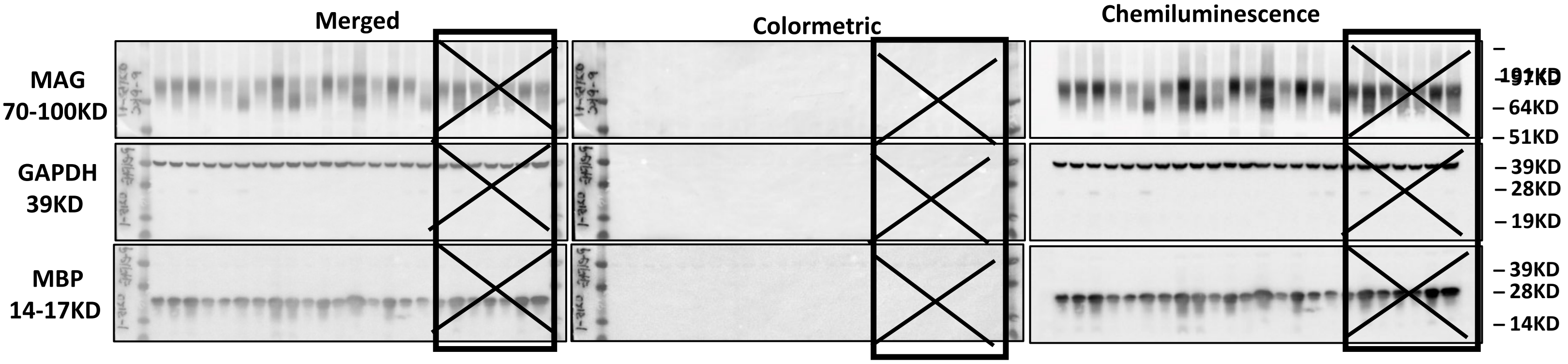

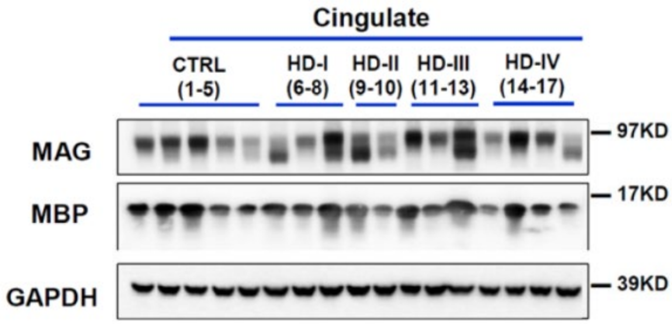

0215-3 p-PKCε - GAPDH - MBP

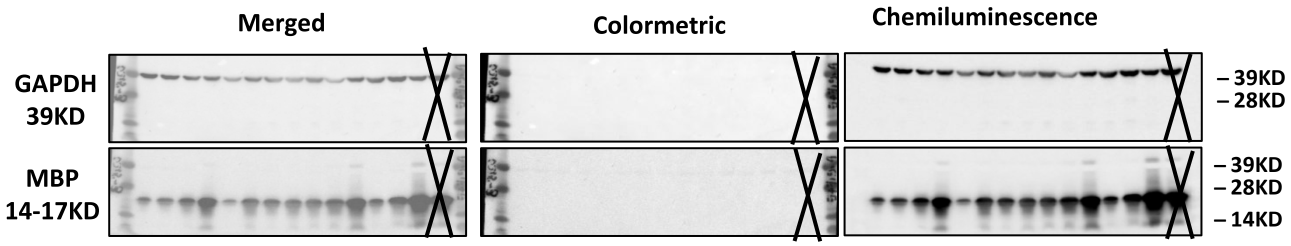

0215-4 MAG – GAPDH

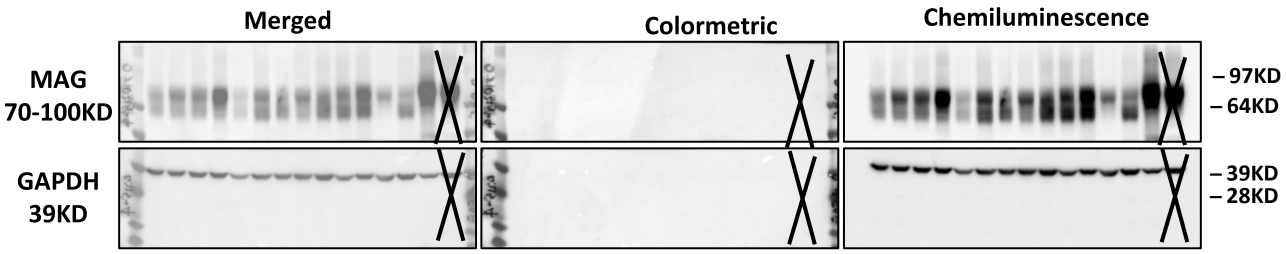

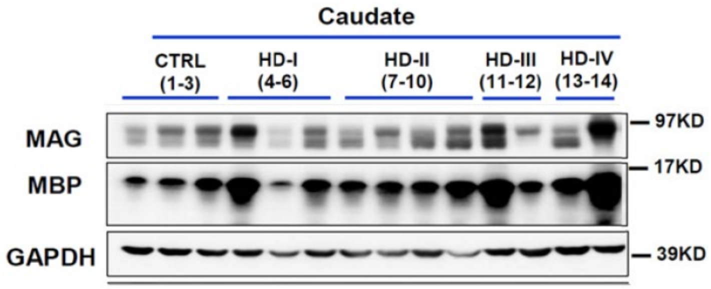

“X” indicates samples that are of a different condition not related to the study

Figure S8B - Gels 0301 – 0304, SGK1, ~50-60KD

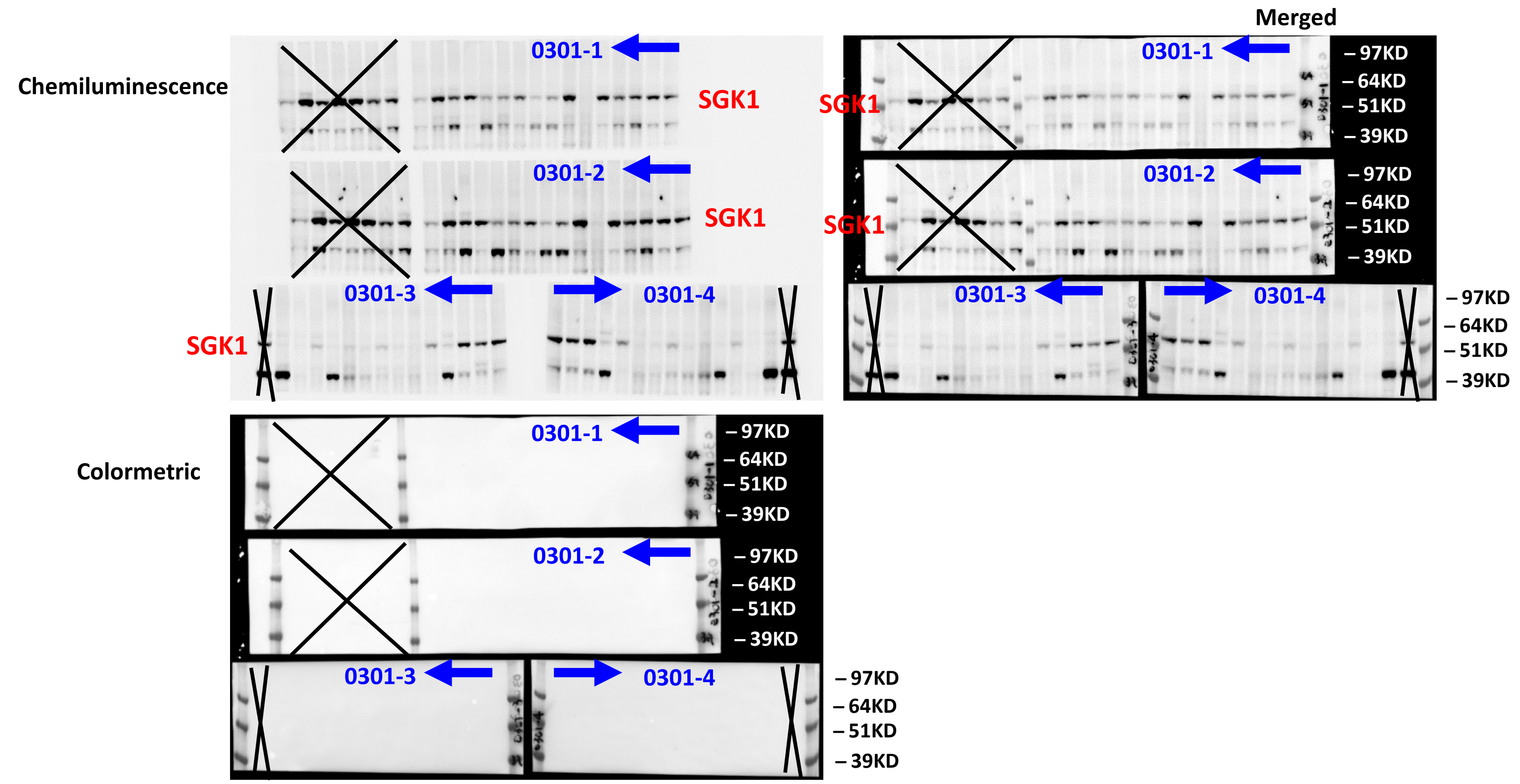

0301 – 0304, GAPDH, 39KD – used for calculating relative abundance of protein – presented in quantifications

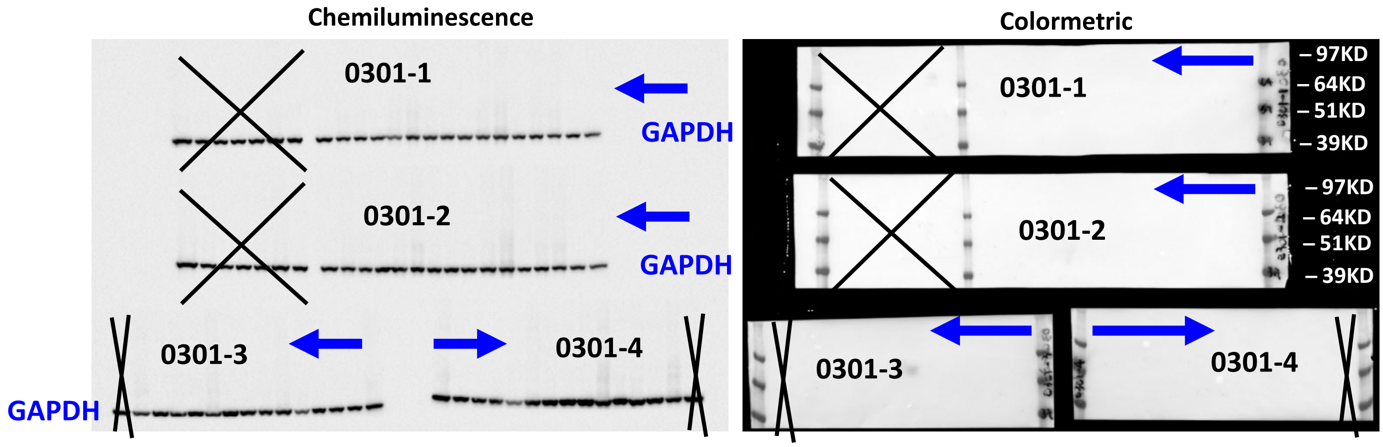

Gels 0301-1 - Cingulate
Gels 0301-3 - Caudate

The arrow indicates the direction of samples with respect to those in the figure – right pointing arrow means same order, left pointing arrow indicates reverse order

"X" indicates samples of a different condition not related to the study

Gels 0301-1 and 0301-2 are duplicates
Gels 0301-3 and 0301-4 are duplicates

Figure S9e – Htt (5492) – Prkce – Revert (cortex & striatum)

5492 and Prkce insolb Cortex R6/2

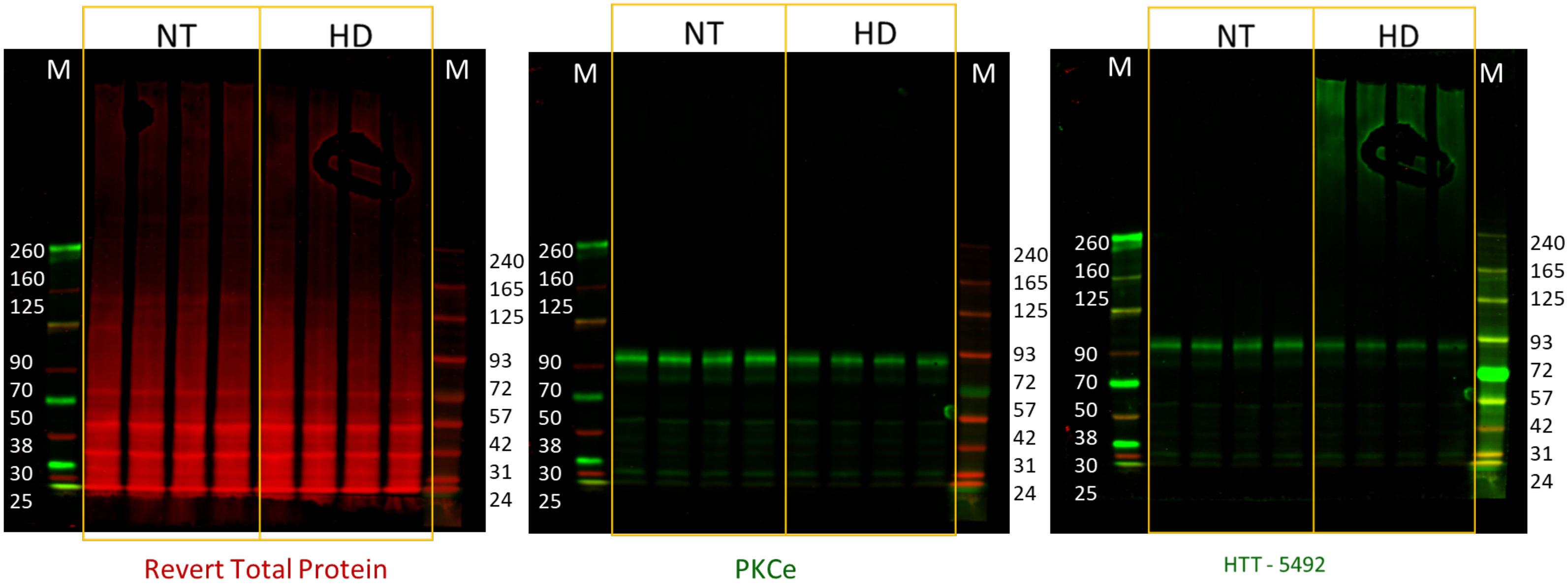

5492 and Prkce insolb Striatum R6/2

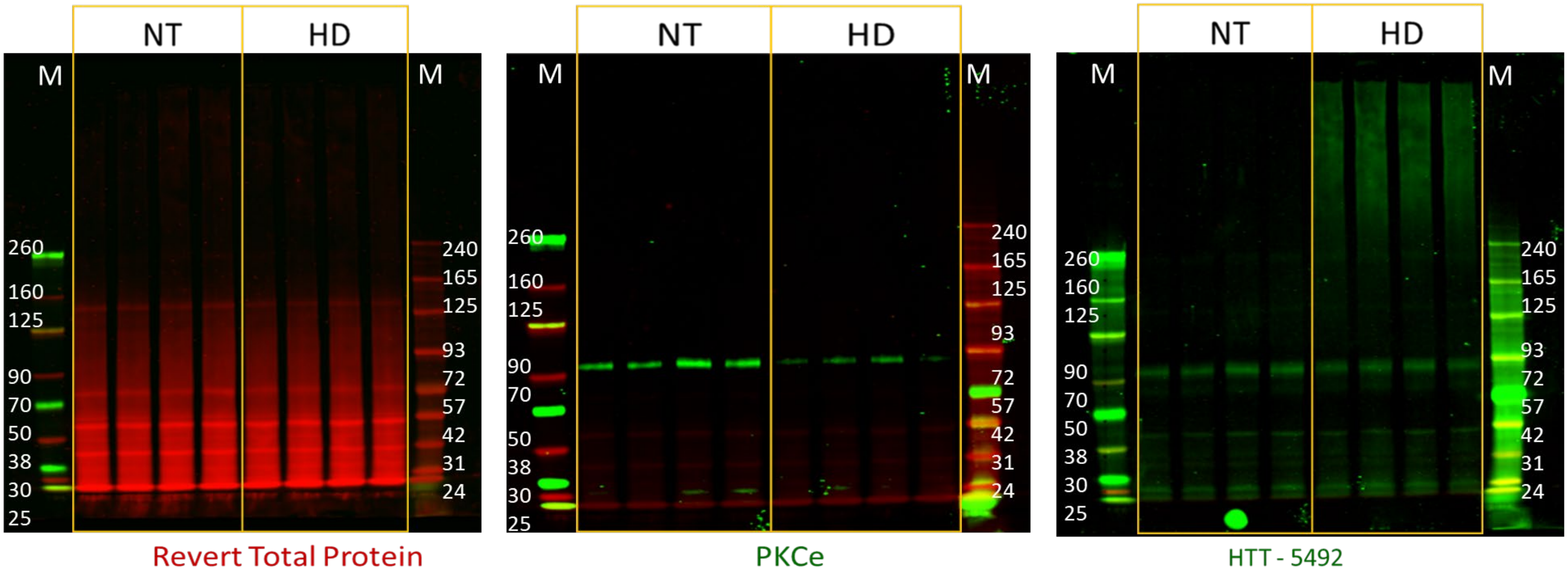

Figure S9e - Dgkb – Revert (cortex & striatum)

DGKB Cortex R6/2

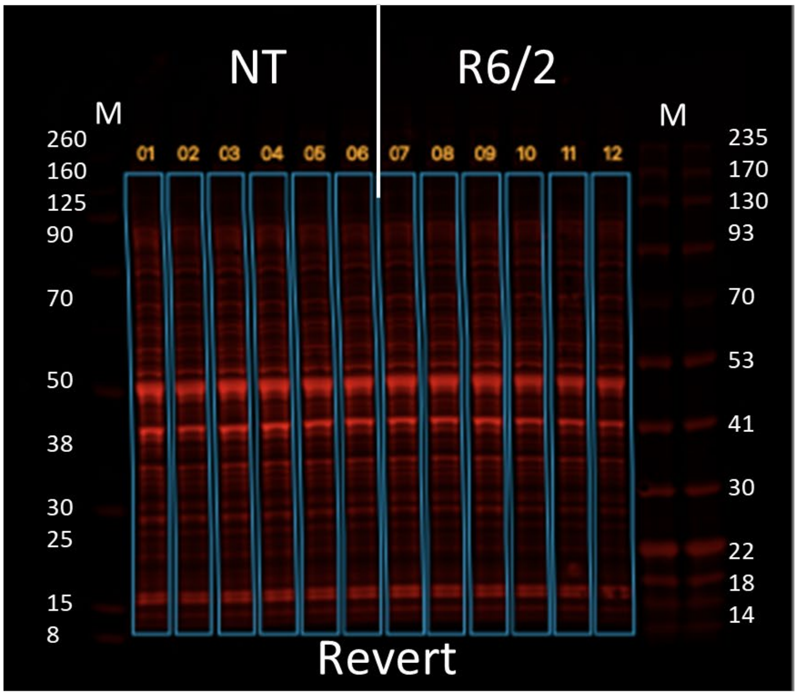

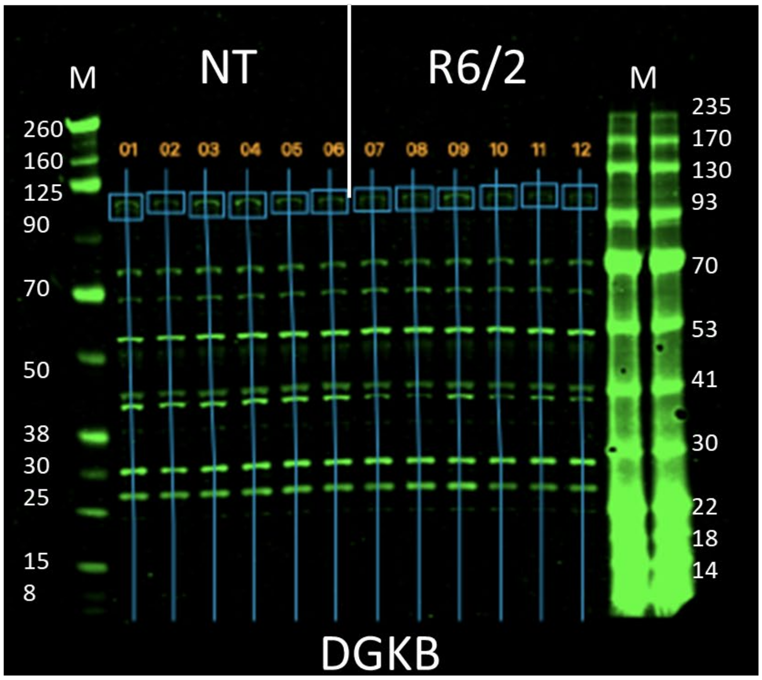

DGKB Striatum R6/2

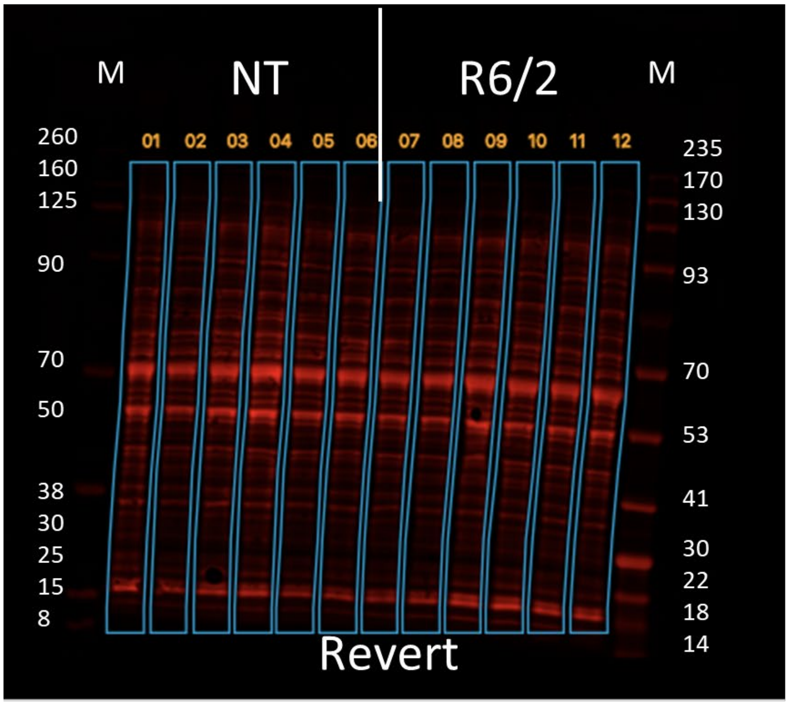

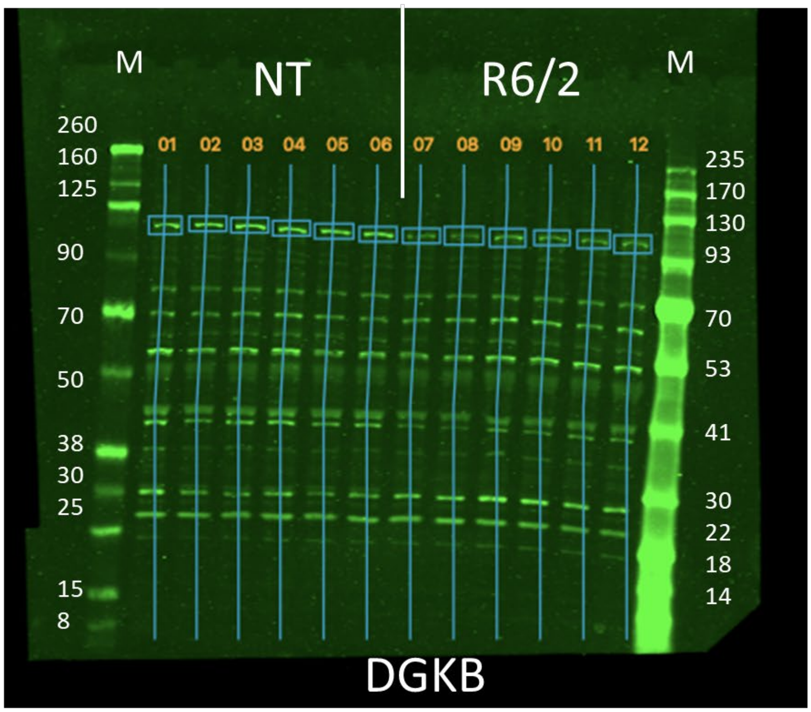

Figure S9e - GPI – Revert (cortex & striatum)

Gpi1 Cortex R6/2

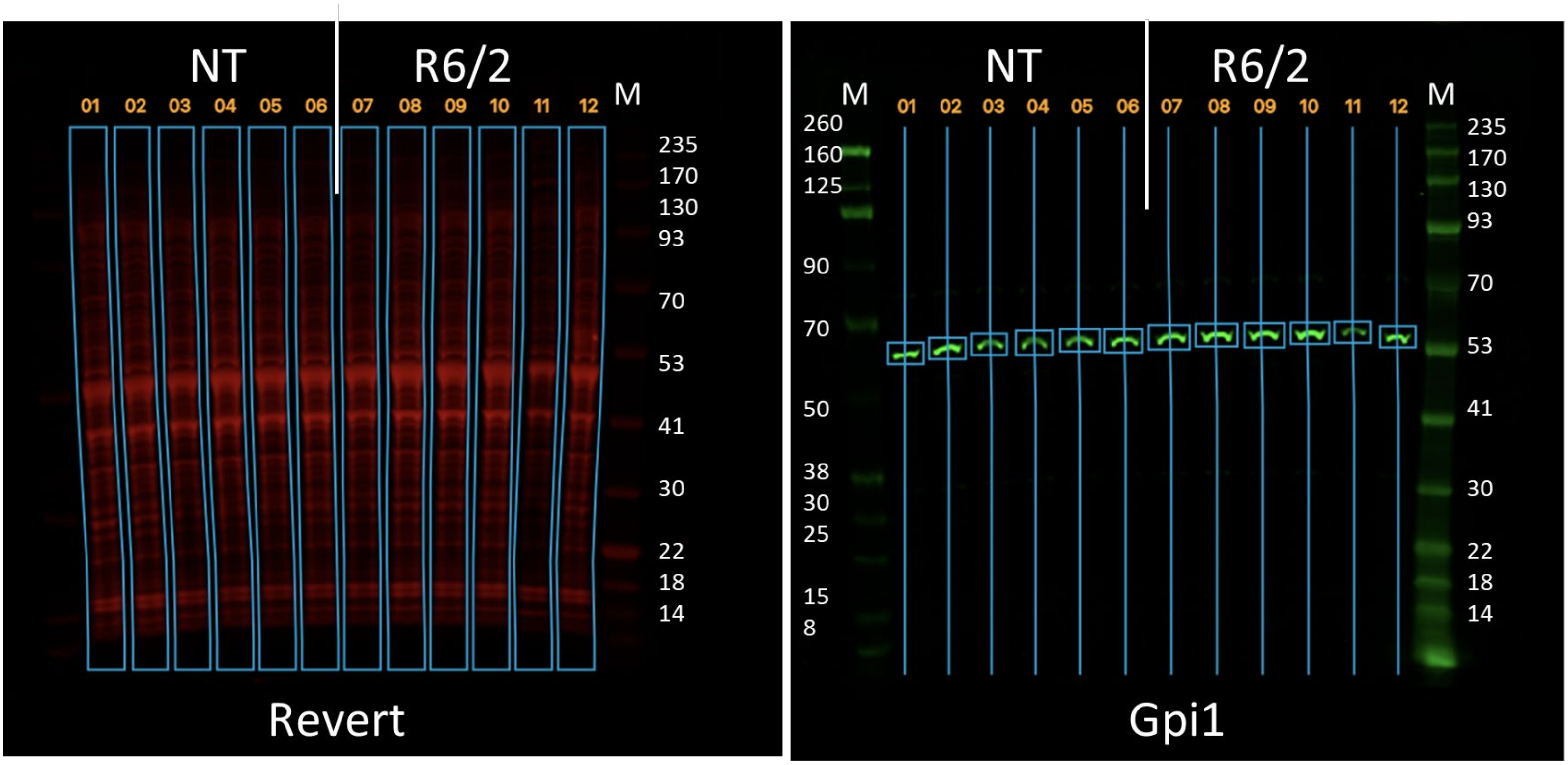

Gpi1 Striatum R6/2

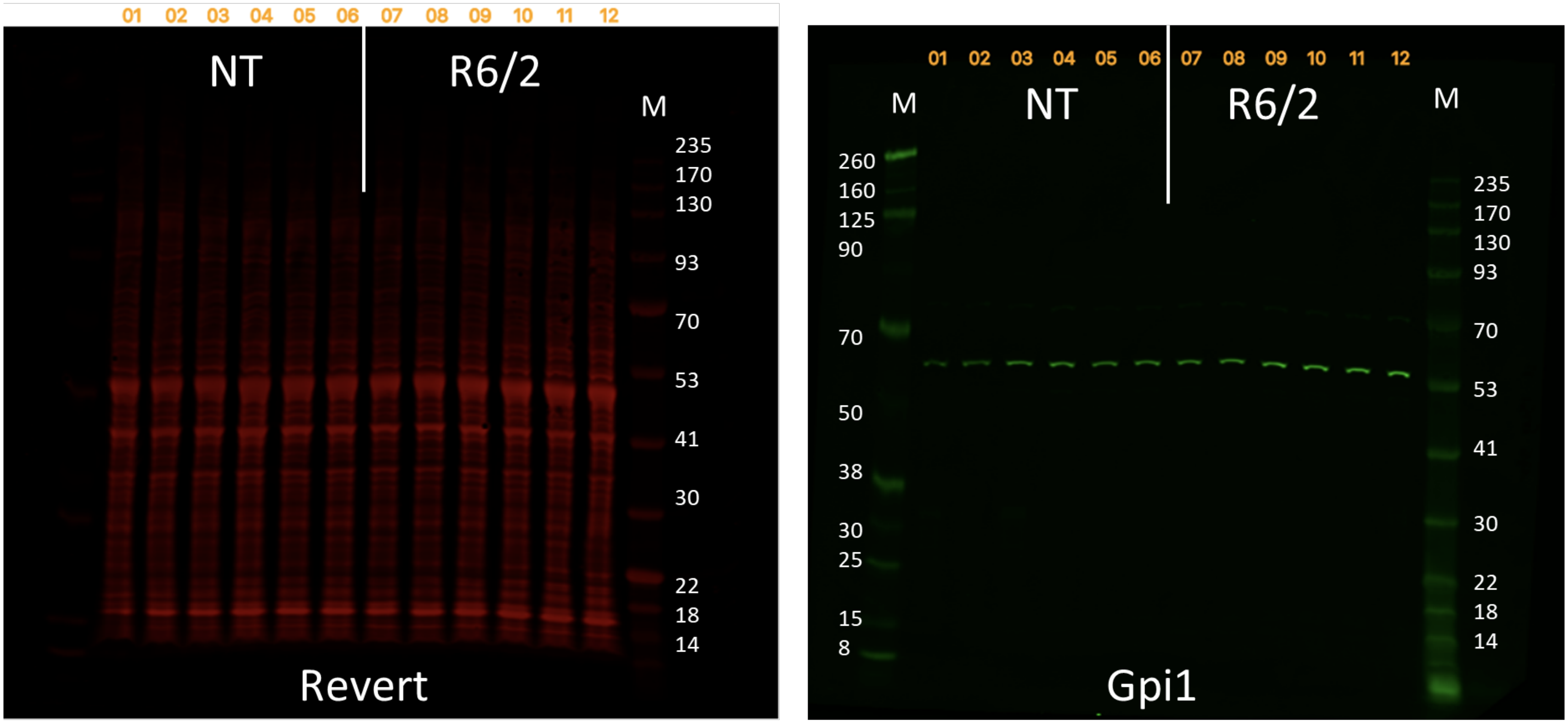

Figure S9e – Sgk1 – Revert (cortex & striatum)

Sgk1 Cortex R6/2

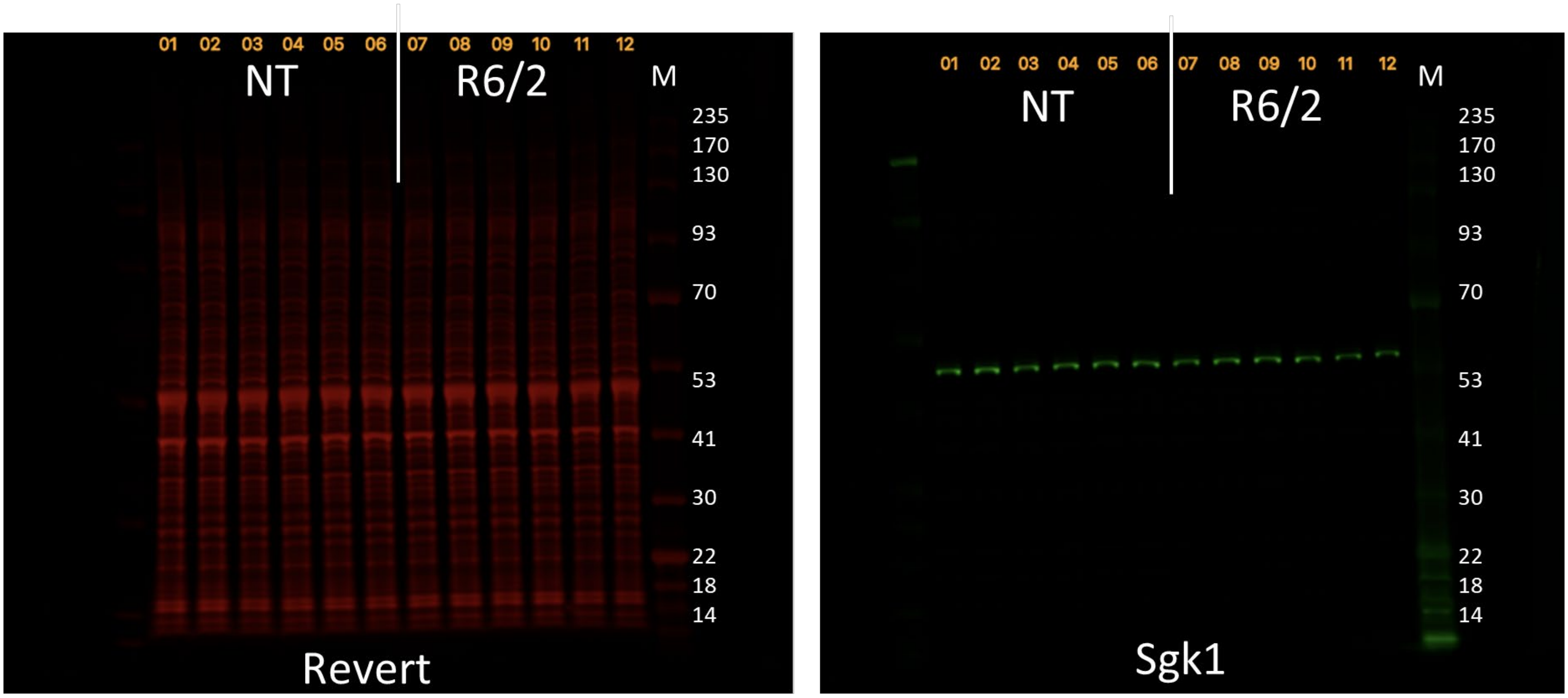

Sgk1 Striatum R6/2

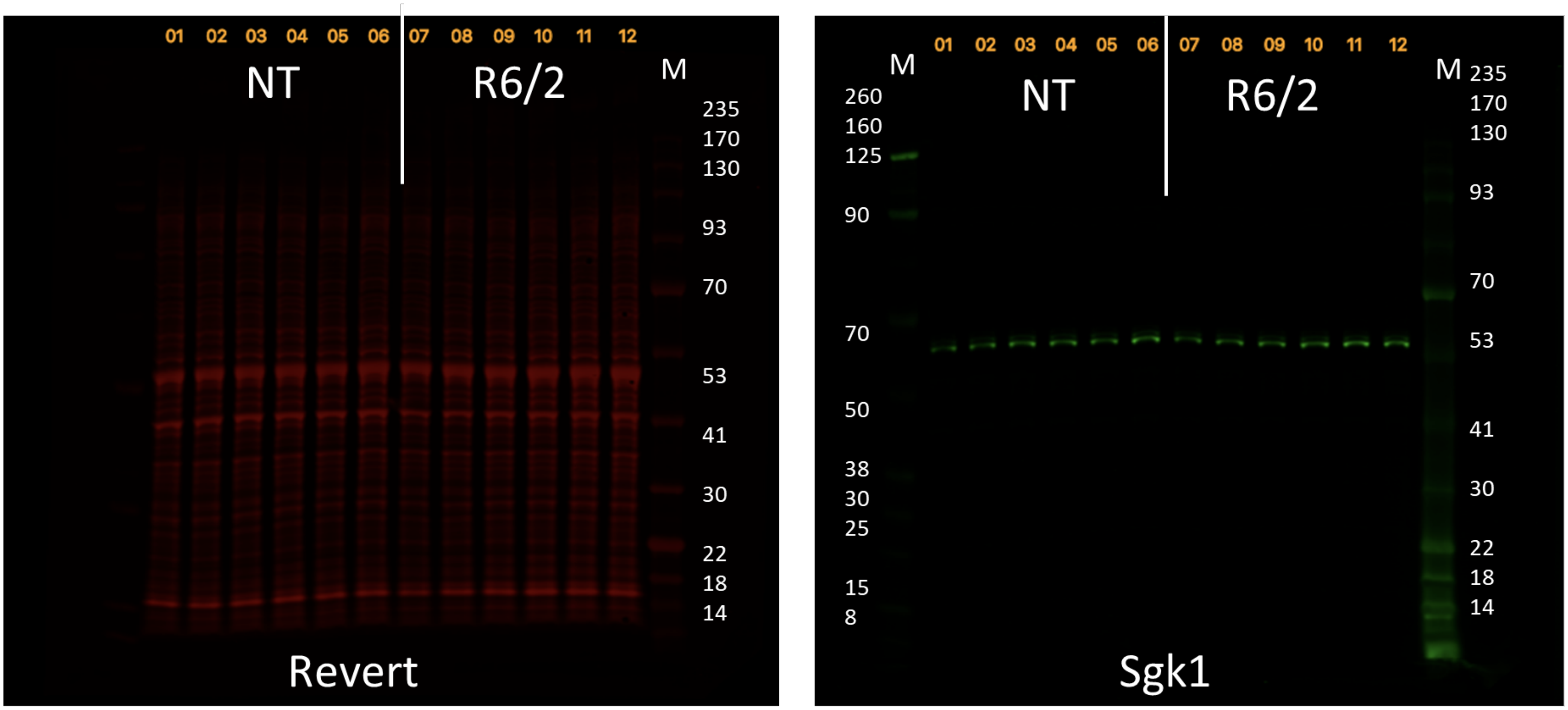

Supplement: Supplementary file 16 — Source Data [file 41467_2022_35388_MOESM16_ESM.zip › Source Data/Source Data File 1 15NOV2022 NCOMMS-21-24870B_Thompson v2.pdf]
